# SEQuential tUnneled Catheters and Infective Events in haemodiaLysis: A Five-Year Retrospective Cohort Study (SEQUEL Study)

**DOI:** 10.1371/journal.pone.0362091

**Published:** 2026-11-04

**Authors:** Shing Shen Bay, Suek Xuan Pon, Xin Yu See, Soo Ying Yew, Elisya Liyana Abdullah, Khai Vern Poon, Chee Keong Thye, Shian Feng Cheng, Yee Wan Lee, Maisarah Jalalonmuhali, Wan Ahmad Hafiz Wan Md Adnan, Chye Chung Gan

**Affiliations:** 1 Nephrology Unit, Medical Department, Faculty of Medicine, Universiti Malaya, Kuala Lumpur, Malaysia; 2 Nephrology Unit, Universiti Malaya Medical Centre, Kuala Lumpur, Malaysia; Universidad Santa Paula, COSTA RICA

## Abstract

**Background:**

Tunnelled-cuffed catheters (TCCs) are widely used for haemodialysis access when permanent vascular access is unavailable. Internal jugular vein (IJV) placement is guideline-preferred, but femoral vein (FV) catheters are frequently used when upper venous access is limited. Whether femoral access confers a higher infection risk than catheter sequence number remains unclear. This study evaluated these two competing determinants of catheter-related bloodstream infection (CRBSI).

**Methods:**

This single-center retrospective cohort study included all TCC insertions at a tertiary nephrology unit between January 2016 and December 2021. The primary analysis examined CRBSI incidence per 1000 catheter-days stratified by catheter sequence number (first, second, third+ insertion per patient). Secondary analyses compared femoral versus internal jugular TCCs overall and within sequence strata. A registry-index cohort (patients with no documented prior TCC before January 2016, n = 135) was used to reduce, though not eliminate, sequence-related confounding in the site comparison; given only 24 CRBSI events, this analysis is exploratory and a clinically meaningful site difference cannot be excluded. Kaplan–Meier methods with log-rank testing were used for time-to-event outcomes. A cluster-robust marginal Cox model was used to account for repeated catheter insertions within patients, adjusting for sequence number, insertion site, age, sex, insertion setting, and exchange from a non-tunnelled catheter.

**Results:**

Among 626 TCC insertions in 339 patients (389 FV, 237 IJV), contributing 98,727 catheter-days, CRBSI incidence escalated with catheter sequence: 1.17 per 1000 catheter-days for first insertions, rising to 1.98 for second and 2.49 for third-or-more insertions. Notably, by the second insertion, CRBSI rates were similar between FV and IJV access (31.7% each). In the index cohort (n = 135; FV = 95, IJV = 40), CRBSI-free survival did not differ significantly between sites (log-rank p = 0.340), nor did catheter survival (p = 0.950) or patient survival (p = 0.487). In the all-TCC cohort, after adjustment for catheter sequence number, age, sex, insertion setting, and exchange from a non-tunnelled catheter using a cluster-robust marginal Cox model, femoral access remained associated with a higher, though attenuated, CRBSI hazard after adjustment for the measured covariates (HR 1.43, 95% CI 1.02–2.01; p = 0.039), indicating a modest residual site hazard beyond the sequence effect.

**Conclusions:**

Repeated catheterization was independently associated with increasing CRBSI risk, and catheter sequence number was the strongest predictor in the fully adjusted model. The femoral site effect was substantially attenuated after adjustment for sequence and was not statistically significant in the registry-index cohort analysis, though the wide confidence intervals preclude an equivalence interpretation. IJV access remains preferred; when IJV is unavailable, femoral TCC placement may be an acceptable alternative from an infection-risk perspective, but this must be weighed against thrombotic and limb complications, including deep vein thrombosis, which were not systematically ascertained in this study. Residual confounding cannot be excluded in this single-center retrospective study.

## 1. Introduction

Haemodialysis is the predominant form of renal replacement therapy in many regions, including Malaysia, where incident end-stage renal disease (ESRD) continues to rise [1,2]. When arteriovenous fistulas (AVFs) are unavailable or immature, tunnelled-cuffed catheters (TCCs) are widely used [3,4]. The internal jugular vein (IJV) is guideline-preferred over the femoral vein (FV) due to its more direct anatomical course and lower reported complication rates [3,5]; FV access is typically reserved for patients with exhausted upper venous access or when upper limb vasculature must be preserved for future AVF creation.

Despite this conventional preference, the literature comparing femoral and jugular TCCs is inconsistent [6,7]. A key methodological limitation of most comparative studies is the failure to account for prior catheter history. In clinical practice, patients on long-term TCC-based access rarely have only one catheter. By their second or third insertion, patients will have accumulated cumulative central venous instrumentation and potential microbial colonisation, while viable access options have progressively narrowed. Pooling all catheter insertions without accounting for sequence position may systematically disadvantage whichever site is more often used in complex, repeat-catheter patients.

Cumulative catheter exposure and biofilm development are known contributors to line infection, and registry data suggest that repeated central venous instrumentation is associated with higher infection rates [5,8]. Yet no study has directly examined whether CRBSI risk rises with each successive catheter independently of insertion site. Femoral catheters accounted for 70% of index TCC insertions at our centre, driven by a policy of preserving upper-limb vasculature for future fistula creation and a high rate of anatomical IJV unsuitability. This femoral-heavy cohort is particularly susceptible to confounding by case mix, providing a relevant context in which to examine the independent contributions of site and sequence to infection risk.

This study evaluates CRBSI incidence across catheter sequence numbers in a five-year single-center cohort of 626 TCC insertions. The primary objective was to determine whether catheter sequence number is independently associated with escalating CRBSI incidence and whether femoral versus internal jugular access modifies this relationship. To disentangle site from sequence, two analytical populations are used throughout: the all-TCC cohort (all 626 insertions) for the primary sequence analysis and cluster-robust Cox modelling, and a registry-index cohort restricted to patients with no documented prior TCC before January 2016 (n = 135) for a site comparison with reduced within-patient sequence confounding. Secondary objectives were to compare CRBSI-free, catheter, and patient survival between sites in the index cohort and to identify independent predictors of CRBSI by multivariable Cox analysis.

## 2. Methodology

### 2.1. Study design

This was a retrospective cohort study conducted at the Nephrology Unit, Universiti Malaya Medical Centre (UMMC), a 1,617-bed academic tertiary referral hospital. All patients who underwent TCC insertion between 1 January 2016 and 31 December 2021 were included. The all-TCC cohort (all insertions, n = 626) served as the primary population for the sequence-number analysis; the index cohort (first TCC per patient with no documented prior TCC before January 2016, n = 135) was used for a site-comparison analysis with reduced sequence confounding. Each catheter was followed from insertion to removal, death, or end of follow-up. This study is reported in accordance with the STROBE guidelines for observational studies; the completed STROBE checklist is provided as Supporting Information. Data were accessed for research purposes on 31/03/2022.

### 2.2. Ethics statement

This study received approval from the Medical Research Ethics Committee, Universiti Malaya Medical Centre (UMMC) (MREC No. 20211117-10778), and was conducted in accordance with the principles of the Declaration of Helsinki (1975). As this was a retrospective study involving review of existing medical records, individual patient consent was waived by the ethics committee. All data were anonymised prior to analysis; no personally identifiable information was retained in the analysis dataset. All data were stored in password-protected institutional servers with access restricted to the study team.

### 2.3. Catheter insertion protocol

All TCCs were inserted by trained nephrologists using ultrasound guidance and standard aseptic technique. Fluoroscopy was not routinely available; tip position was confirmed by chest radiograph and were unsatisfactory, by referral to interventional radiology. Femoral TCC was used when internal jugular access was unavailable or unsuitable, upper limb vasculature required preservation for fistula creation, or IJV insertion had failed at the same sitting.

### 2.4. Definitions

The index TCC was defined as the first tunnelled-cuffed catheter per patient during the study period (January 2016 to December 2021). Patients with any documented history of TCC insertion prior to January 2016 were excluded, so the index catheter reflects the patient’s first registry-recorded TCC insertion. CRBSI was defined per IDSA criteria: concordant growth from paired blood cultures (catheter and peripheral), or from a catheter tip and peripheral culture [9]. Clinically adjudicated probable CRBSI (without microbiological confirmation) was defined as fever, rigors, or haemodynamic instability occurring in a patient with a TCC, in whom no alternative infectious source was identified after clinical assessment and available microbiological investigations, and who showed clinical improvement following catheter-directed management and/or antimicrobial therapy; cases with a documented non-catheter source of infection were excluded. Culture-negative status was interpreted in the context of antimicrobial exposure before blood-culture collection, where documented. Exit-site and tunnel infections without concurrent bloodstream involvement were not classified as CRBSI and were not included in the primary outcome. The primary outcome was confirmed or clinically adjudicated probable CRBSI events, comprising 131 (85.1%) microbiologically confirmed episodes and 23 (14.9%) clinically adjudicated probable CRBSI episodes; each probable episode was adjudicated against the above criteria by the attending nephrologist and independently reviewed by the lead investigator, for a total of 154 CRBSI events. Pre-culture antibiotic exposure was not systematically recorded and is acknowledged as a limitation of adjudicating culture-negative episodes (Section 4, Limitations). A pre-specified sensitivity analysis restricted to microbiologically confirmed events is reported in Section 3.9. Differential time-to-positivity and single peripheral culture results were evaluated per the attending clinician and classified according to IDSA criteria; where catheter tip culture was unavailable, concordant peripheral paired cultures were used. CRBSI incidence was expressed per 1000 catheter-days. Catheter dwell time was the interval from insertion to removal or censoring. Patient survival was time from insertion to all-cause death. Early complications occurred within 48 hours; late complications thereafter [3].

### 2.5. Data collection

#### 2.5.1. Inclusion and exclusion criteria.

The all-TCC cohort included all TCC insertions at UMMC between 1 January 2016 and 31 December 2021 in patients aged 18 years or older, regardless of prior TCC history, provided the catheter was inserted at this centre for haemodialysis and key data (catheter site, insertion date, and outcome) were available. Catheters inserted at another centre, for an indication other than haemodialysis, or with missing key data were excluded. Patients lost to follow-up immediately after insertion were also excluded. The index cohort was a subset of the above, restricted to patients with no documented prior TCC insertion before January 2016 (i.e., registry sequence number 1), reducing but not eliminating sequence-related confounding in the site comparison. Patients with any prior TCC history before the study period were excluded from the index cohort but remained eligible for the all-TCC cohort with their registry sequence number assigned from their first recorded insertion onward. Non-tunnelled dialysis catheters (NTDCs) were excluded from analysis. Unlike TCCs, NTDCs lack a subcutaneous tunnel and Dacron cuff, which serve as mechanical barriers to extraluminal bacterial migration — a distinct pathophysiological route for CRBSI. Inclusion of NTDCs would introduce heterogeneity in infection mechanism that could not be adequately addressed through statistical adjustment alone.

### 2.6. Statistical analysis

All statistical analyses were performed using Python. The primary analysis quantified CRBSI incidence per 1000 catheter-days stratified by catheter sequence number (first, second, third, and fourth-or-more insertion per patient), assigned chronologically per patient within the registry. For patients with TCC insertions prior to January 2016, sequence numbers in the registry may underestimate true lifetime sequence, biasing reported sequence HRs toward the null; the analysis is therefore conservative in this respect. CRBSI rates were further cross stratified by sequence position and access site to test whether site modifies the sequence effect. All hypothesis tests were two-sided (α = 0.05); secondary and subgroup analyses are exploratory. Missing data were rare: one record missing the CRBSI outcome was excluded from event-based analyses (Cox model n = 625); three records with missing INR were excluded from INR comparisons only. No imputation was performed.

Baseline and procedural characteristics were described separately for the index cohort (first TCC per patient, n = 135) and the all-TCC cohort (all 626 insertions). Continuous variables are presented as median with interquartile range (IQR); categorical variables as counts and column percentages. Between-group comparisons (FV vs IJV) used the Mann–Whitney U test for continuous variables, Fisher’s exact test for 2 × 2 tables, and the Pearson chi-square test for larger contingency tables. To examine whether CRBSI events were preferentially followed by femoral re-catheterisation, a one-sample binomial test was applied against the overall femoral-to-IJV registry ratio.

CRBSI incidence density was calculated as the number of events divided by total catheter-days at risk, expressed per 1000 catheter-days; exact 95% confidence intervals were estimated using the Poisson distribution. Time-to-event outcomes (CRBSI-free survival, catheter survival, and all-cause patient survival) were estimated using the Kaplan–Meier product-limit method. Catheter removal following arteriovenous fistula maturation was treated as a non-informative censoring event in the catheter survival analysis, as it represents successful transition to permanent access rather than catheter failure. A formal Fine–Gray competing risks model was not applied because the primary interest was the cause-specific CRBSI hazard; most competing events (AV fistula maturation) are clinically unrelated to the CRBSI process and do not violate the non-informative censoring assumption for the cause-specific analysis. Cumulative incidence estimates treating non-CRBSI removal and death as competing events are reported descriptively in the supplementary material. Year of insertion was explored as a covariate in the Cox model; it was not independently associated with CRBSI after adjustment for sequence number and was not retained in the final model. Between-group differences in survival distributions were evaluated using the log-rank test. The median time from catheter insertion to first CRBSI was summarised with IQR to characterise the temporal pattern of infection onset within each cohort.

Multivariable analysis used a Cox proportional hazards model in the all-TCC cohort, with within-patient correlation handled by the Lin–Wei–Ying marginal approach with robust sandwich variance estimation, treating each patient as a cluster [10,11]. Covariates were pre-specified: catheter insertion site (FV vs IJV), sequence number (first, second, third-or-more), patient age, sex, insertion setting (inpatient vs outpatient), and exchange from a non-tunnelled catheter. Hazard ratios (HR) are reported with 95% CIs. The proportional hazards assumption was verified by scaled Schoenfeld residuals [12]. The exchange variable was retained regardless of significance given its biological plausibility (pre-existing biofilm from an antecedent non-tunnelled line); its exclusion did not alter site or sequence HRs. Diabetes mellitus was not independently associated with CRBSI (chi-square p = 0.468) and was excluded; a sensitivity model including it yielded unchanged results. Patients contributing both femoral and jugular catheters (n = 47) contributed separate observations to each site-stratified Kaplan–Meier curve.

Supplementary analyses included: year-on-year CRBSI incidence trends; quantification of within-patient repeated catheterisations and access-site crossover; and tabulation of catheter removal reasons, salvage rates, and median time to first CRBSI. A sensitivity analysis in the index cohort (n = 135) used univariable log-rank testing and exact Poisson incidence densities; given only 24 CRBSI events, no multivariable model was fitted, and this analysis is considered exploratory. Three pre-specified all-TCC sensitivity analyses were performed: (1) restricting to the 131 microbiologically confirmed CRBSI events only (excluding 23 clinically adjudicated probable CRBSI episodes); (2) adding ethnicity to the Cox model; and (3) restricting to patients with three or fewer insertions (n = 310 patients, 467 catheters). Analysis scripts are available from the corresponding author.

## 3. Results

### 3.1. Study population

Between January 2016 and December 2021, 626 TCC insertions were identified: 389 (62.1%) femoral vein (FV-TCC) and 237 (37.9%) internal jugular veins (IJV-TCC). (Fig 1).

**Fig 1:**
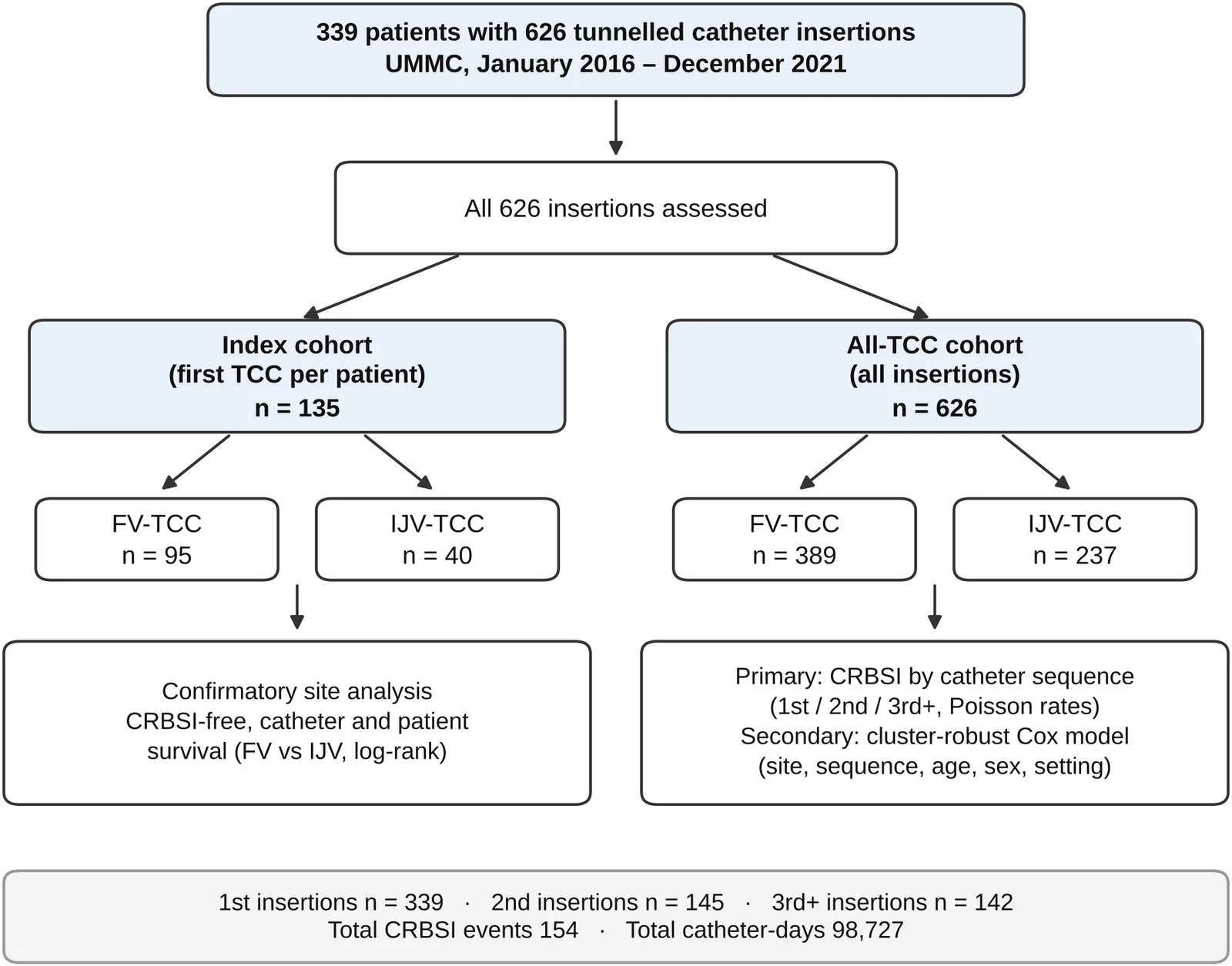
Study cohort flowchart — SEQUEL study (Jan 2016–Dec 2021). A total of 339 unique patients contributed 626 catheter insertions. The all-TCC cohort (n = 626) was the primary dataset for sequence-stratified incidence analysis and cluster-robust Cox modelling. The registry-index cohort (n = 135, patients with no documented prior TCC before January 2016) was used for the site-comparison analysis with reduced within-patient sequence confounding.

Of these, 135 patients contributed registry-index TCC insertions (patients with no documented prior TCC before January 2016) forming the site-comparison cohort with reduced sequence confounding: 95 (70.4%) FV-TCC and 40 (29.6%) IJV-TCC. The remaining 491 catheters were subsequent registry insertions.

### 3.2. Baseline characteristics

Baseline characteristics were broadly comparable between index FV-TCC and IJV-TCC groups, apart from ethnic distribution, which differed significantly (p = 0.033). Cardiometabolic comorbidity was near-universal in both groups, with diabetes as the predominant ESRD cause. In the all-TCC cohort, femoral catheters were more often inserted in inpatients (61.7% vs 33.3%; p < 0.001) and had a shorter median dwell time than IJV catheters (66 vs 137 days; p < 0.001). Detailed baseline and procedural characteristics are shown in Table 1.

**Table 1:** Baseline characteristics, procedural data, and outcomes by access site and cohort.

Variable	Index FV (n = 95)	Index IJV (n = 40)	p	All FV (n = 389)	All IJV (n = 237)	p
**Demographics**						
Age, years, median (IQR)	62.0 (53.0–68.0)	58.5 (49.3–72.0)	0.975	61.0 (54.0–68.0)	60.0 (53.0–69.0)	0.705
Male sex, n (%)	55 (57.9%)	22 (55.0%)	0.849	207 (53.2%)	108 (45.6%)	0.070
Female sex, n (%)	40 (42.1%)	18 (45.0%)		182 (46.8%)	129 (54.4%)	
**Ethnicity, n (%)**			**0.033**			**0.014**
Malay	56 (58.9%)	17 (42.5%)		228 (58.6%)	109 (46.0%)	
Chinese	17 (17.9%)	15 (37.5%)		74 (19.0%)	64 (27.0%)	
Indian	22 (23.2%)	7 (17.5%)		86 (22.1%)	62 (26.2%)	
Other	0 (0.0%)	1 (2.5%)		1 (0.3%)	2 (0.8%)	
**Setting at insertion, n (%)**						
Inpatient	41 (43.2%)	22 (55.0%)	0.258	240 (61.7%)	79 (33.3%)	<0.001
Outpatient	54 (56.8%)	18 (45.0%)		149 (38.3%)	158 (66.7%)	
**Comorbidity & medications**						
Premorbid (DM/HTN/Dyslip), n (%)	89 (93.7%)	37 (92.5%)	0.724	365 (93.8%)	223 (94.1%)	1.000
Antiplatelet/anticoagulant use, n (%)	47 (49.5%)	15 (37.5%)	0.257	176 (45.2%)	105 (44.3%)	0.868
**Cause of ESRD, n (%)**			**0.353**			**0.579**
Diabetes mellitus	79 (83.2%)	28 (70.0%)		327 (84.1%)	183 (77.2%)	
Hypertension	5 (5.3%)	4 (10.0%)		29 (7.5%)	28 (11.8%)	
Primary GN	3 (3.2%)	3 (7.5%)		10 (2.6%)	7 (3.0%)	
SLE	1 (1.1%)	2 (5.0%)		4 (1.0%)	3 (1.3%)	
Unknown	4 (4.2%)	1 (2.5%)		9 (2.3%)	9 (3.8%)	
Other	3 (3.2%)	2 (5.0%)		10 (2.6%)	7 (3.0%)	
**Procedural**						
Exchange from non-tunnelled, n (%)	2 (2.1%)	0 (0.0%)	1.000	111 (28.5%)	57 (24.1%)	0.228
Right-sided position, n (%)	71 (74.7%)	30 (75.0%)	1.000	272 (69.9%)	171 (72.2%)	0.587
Catheter dwell, days, median (IQR)	51 (26.5–115)	68 (28–202)	0.269	66 (29–131)	137 (48–284)	<0.001
Total catheter-days	9527	6409		49531	49196	
**Baseline laboratory**						
Platelets (×10^9^/L), median (IQR)	248 (182–308)	236 (165–296)	0.345	229 (179–284)	238 (174–300)	0.390
INR, median (IQR)	1.10 (1.00–1.20)	1.00 (1.00–1.10)	0.097	1.10 (1.00–1.20)	1.00 (1.00–1.10)	0.002
**Complications & outcomes**						
Early bleeding, n (%)	4 (4.2%)	3 (7.5%)	0.422	22 (5.7%)	16 (6.8%)	0.607
Late bleeding, n (%)	10 (10.5%)	3 (7.5%)	0.755	25 (6.4%)	27 (11.4%)	0.036
Any peri-procedural complication, n (%)	4 (4.2%)	3 (7.5%)	0.794	25 (6.4%)	17 (7.2%)	0.743
CRBSI, n (%)	18 (18.9%)	6 (15.0%)	0.806	103 (26.5%)	51 (21.5%)	0.181
Death during follow-up, n (%)	14 (14.7%)	11 (27.5%)	0.093	63 (16.2%)	48 (20.3%)	0.198

IQR, interquartile range. p-values compare FV vs IJV within each cohort: Mann–Whitney U for continuous variables; Fisher’s exact test for 2 × 2 categorical; chi-square for larger contingency tables. Index cohort = patients with no documented prior TCC before January 2016 (registry sequence number 1). All-TCC cohort = every TCC insertion in the registry; patient-level clustering addressed in multivariable analysis using cluster-robust variance estimation. CRBSI incidence density (per 1000 catheter-days, with exact-Poisson 95% confidence intervals) is reported separately in Table 3. Interpretation of the late-bleeding rate difference is discussed in Section 3.6.

### 3.3. Reason for femoral TCC

In the index cohort, the most common indication for TCC insertion was initiation of haemodialysis (49, 36.3%), followed by vascular access thrombosis (46, 34.1%), catheter blockage (15, 11.1%), and infection of a prior non-tunnelled catheter requiring removal (9, 6.7%). These latter categories reflect indications leading to the patient’s index TCC insertion, not a prior TCC. For example, blockage of a previous non-tunnelled or temporary catheter necessitated the index tunnelled insertion.

Among patients who underwent femoral TCC insertion, the most frequent reasons were the absence or unsuitability of internal jugular access (48.4%) and the need to preserve upper limb vasculature for future arteriovenous fistula creation (Table 2). Notably, 44% of index femoral insertions followed a failed IJV attempt at the same sitting, suggesting femoral access was often a necessary rescue rather than a planned first choice. Only 1 of 95 (1.1%) index femoral TCCs, and 2 of 389 (0.5%) femoral TCCs in the all-TCC cohort, were placed as a primarily planned femoral approach unrelated to IJV unavailability or failure (reason: patient preference); the remaining 92.3% of all femoral insertions followed absent, unsuitable, or failed IJV access. The femoral group is therefore best understood as a predominantly rescue-access population rather than a group independently assigned to femoral placement, and site comparisons should be interpreted accordingly.

**Table 2:** Documented indications for femoral vein TCC insertion.

Reason for femoral TCC	Index cohort (n = 95)	All cohort (n = 389)
Preserve vessels for AVF/BCF	5 (5.3%)	26 (6.7%)
No IJV access	46 (48.4%)	210 (54.0%)
Failed IJV TCC attempt	42 (44.2%)	149 (38.3%)
Patient’s choice	1 (1.1%)	2 (0.5%)
Rest fistula	1 (1.1%)	1 (0.3%)
Femoral access as initial planned approach (no prior IJV attempt or indication documented)	0 (0.0%)	1 (0.3%)

Denominator = total femoral TCC insertions in each cohort. Categories are mutually exclusive and adjudicated from procedural records. “No IJV access” indicates pre-procedural assessment of unsuitable or unavailable IJV (anatomical, thrombotic, or prior scarring); “Failed IJV TCC attempt” indicates an unsuccessful IJV insertion attempt at the same sitting before proceeding to femoral access. These two categories were not double counted.

### 3.4. CRBSI incidence by catheter sequence number

Among all 626 TCC insertions, CRBSI incidence per 1000 catheter-days escalated with sequence: 1.17 for first catheters, 1.98 for second, and 2.49 for third-or-more insertions. (Fig 2).

**Fig 2:**
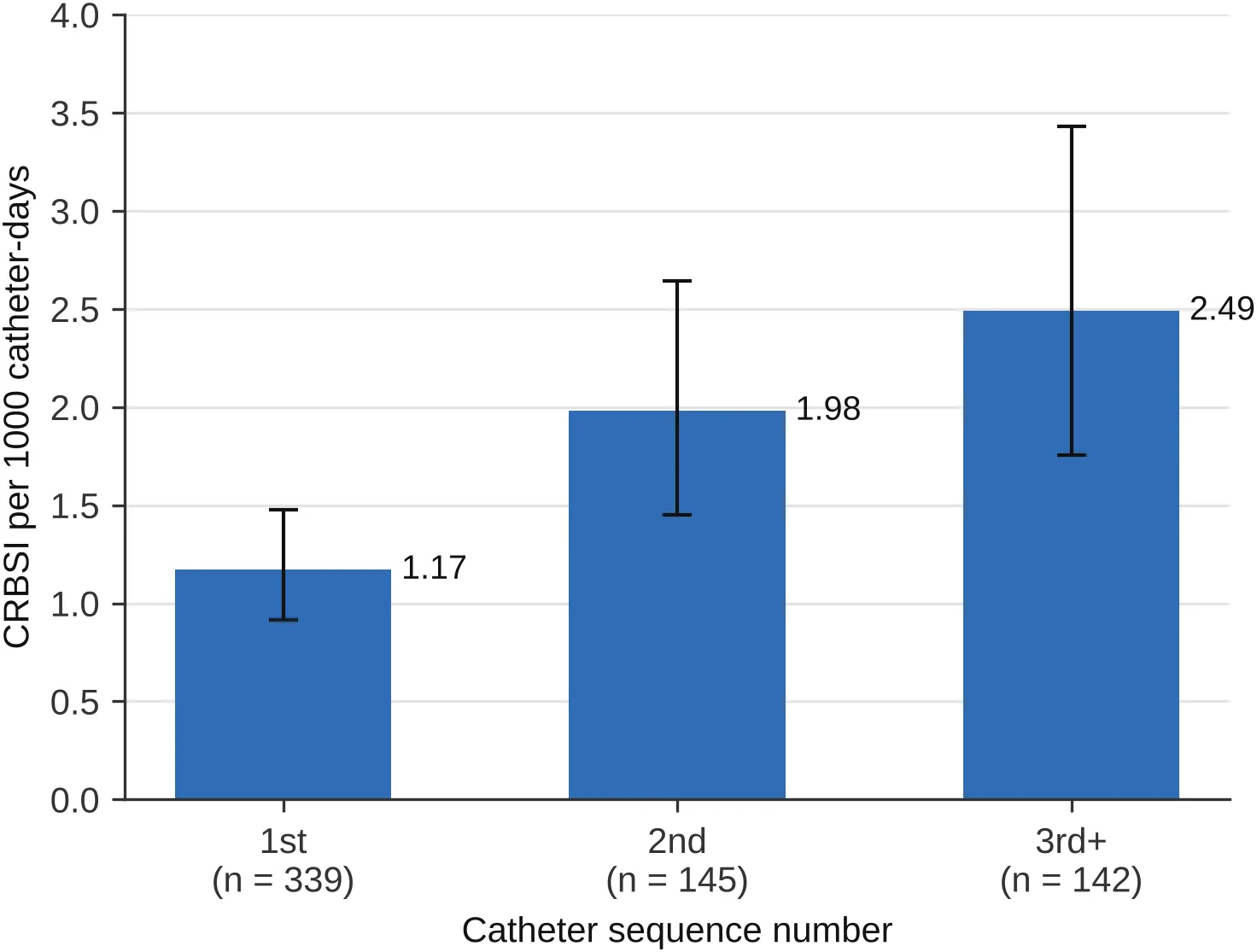
CRBSI incidence rate by catheter sequence number (All-TCC cohort, n = 626). CRBSI rate per 1000 catheter-days: 1st insertion (n = 339, k = 71): 1.17; 2nd insertion (n = 145, k = 46): 1.98; 3rd+ insertion (n = 142, k = 37): 2.49. Error bars = 95% exact Poisson confidence intervals. This escalating pattern is the primary finding of the SEQUEL study.

Cross-stratification by sequence and site showed that while first-catheter CRBSI rates differed numerically (FV-TCC: 1.67/1000 catheter-days, 23.7%; IJV-TCC: 0.83/1000 catheter-days, 18.1%), rates had converged by the second insertion (FV: 2.07, IJV: 1.78 per 1000 catheter-days; proportions 31.7% each). This convergence suggests that the modest first-insertion site difference is outweighed by the cumulative burden of repeated catheterisation from the second insertion onward (Fig 3).

**Fig 3:**
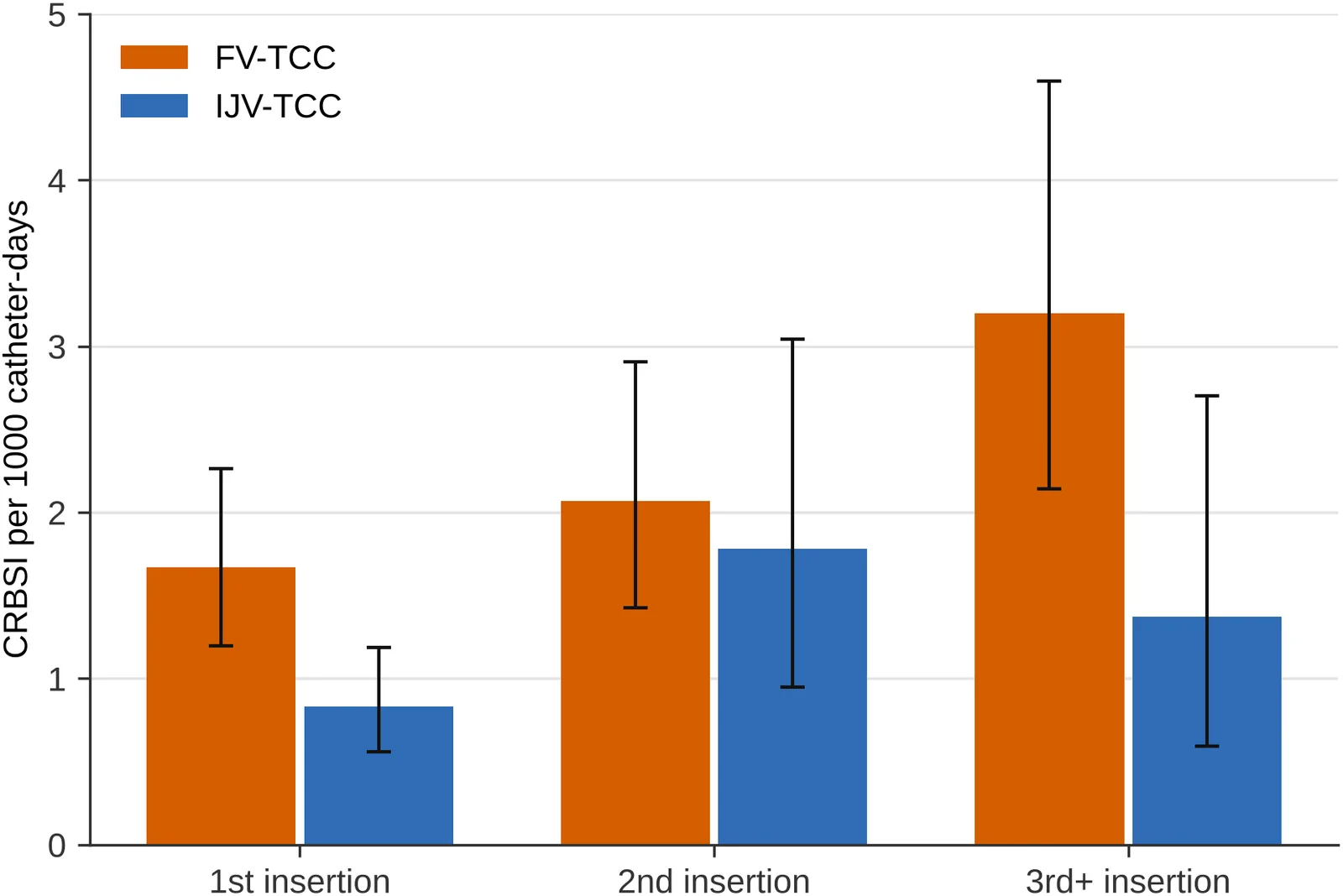
CRBSI incidence by catheter sequence number and access site (All-TCC cohort). CRBSI incidence per 1000 catheter-days stratified by catheter sequence number and access site. At first insertion: FV 1.67/1000 catheter-days, IJV 0.83/1000 catheter-days. At second insertion: FV 2.07, IJV 1.78/1000 catheter-days. Error bars = 95% exact Poisson CI.

Of 339 patients in the registry, 194 (57.2%) required only one TCC during the study period; 145 (42.8%) underwent two or more insertions and 70 (20.6%) required three or more. Patients with repeated catheterisations accounted for 432 of 626 insertions (69.0%) and drove the majority of CRBSI events. Of note, while FV and IJV rates converge at the second insertion (2.07 vs 1.78/1000 cd), a numerical divergence re-emerges at the third-or-more stratum (FV: 3.20, IJV: 1.37/1000 cd). However, the IJV third-or-more group was small (n = 30, k = 8, 95% CI 0.59–2.70) and this divergence does not reach statistical significance. A plausible explanation is selective vascular exhaustion: patients who still have IJV access at the third insertion are those with less accumulated central venous damage, whereas femoral patients at the same sequence position are likely the most vascularly compromised. This hypothesis cannot be tested within the current dataset but warrants prospective investigation.

### 3.5. Catheter-related bloodstream infection (CRBSI)

The site comparison was a secondary analysis; the sequence findings in Section 3.4 are the primary result. CRBSI occurred in 18 of 95 FV-TCC (18.9%) and 6 of 40 IJV-TCC (15.0%) in the index cohort, and in 103 of 389 FV-TCC (26.5%) and 51 of 237 IJV-TCC (21.5%) in the all-TCC cohort; the unadjusted overall site difference is likely influenced by catheter sequence, within-patient clustering, and the predominantly rescue-access nature of femoral placement (Section 3.4).

When expressed as incidence density, CRBSI rates in the index cohort were 1.89 (95% CI 1.12–2.99) per 1000 catheter-days for FV-TCC and 0.94 (95% CI 0.34–2.04) for IJV-TCC. In the all-TCC cohort, the corresponding rates were 2.08 (1.70–2.52) and 1.04 (0.77–1.36) per 1000 catheter-days, respectively (Table 3). While femoral access is statistically associated with higher CRBSI hazard in the all-TCC cohort, the absolute difference in incidence rates in first-catheter episodes was more modest (1.89 vs 0.94 per 1000 catheter-days; difference 0.95/1000 cd, with overlapping 95% confidence intervals), underscoring the importance of accounting for catheter sequence.

**Table 3:** CRBSI incidence density by cohort and access site.

Cohort / Site	n	CRBSI events	Catheter-days	Rate per 1000 catheter-days (95% CI)
Index cohort – FV-TCC	95	18	9,527	1.89 (1.12–2.99)
Index cohort – IJV-TCC	40	6	6,409	0.94 (0.34–2.04)
All-TCC cohort – FV-TCC	389	103	49,531	2.08 (1.70–2.52)
All-TCC cohort – IJV-TCC	237	51	49,196	1.04 (0.77–1.36)

CRBSI, catheter-related bloodstream infection; 95% CI calculated using the exact Poisson method for incidence density. Index cohort = registry-index TCC insertions (patients with no documented prior TCC before January 2016); All-TCC cohort = every TCC insertion in the registry. Values independently recomputed from the study dataset and match the incidence densities cited in the text.

Kaplan–Meier CRBSI-free survival curves are shown in Fig 4 (index cohort) and Fig 5 (all-TCC cohort). The index cohort did not demonstrate a statistically significant site difference (log-rank p = 0.340); however, with only 24 CRBSI events and wide confidence intervals, this result reflects insufficient power to detect the observed near-2-fold difference in incidence density rather than confirmed equivalence. A significant unadjusted difference was observed in the all-TCC cohort (log-rank p < 0.001); however, this likely partly reflects catheter-sequence confounding and patient clustering, and the site effect was attenuated after adjustment in the cluster-robust Cox model (Section 3.9).

**Fig 4:**
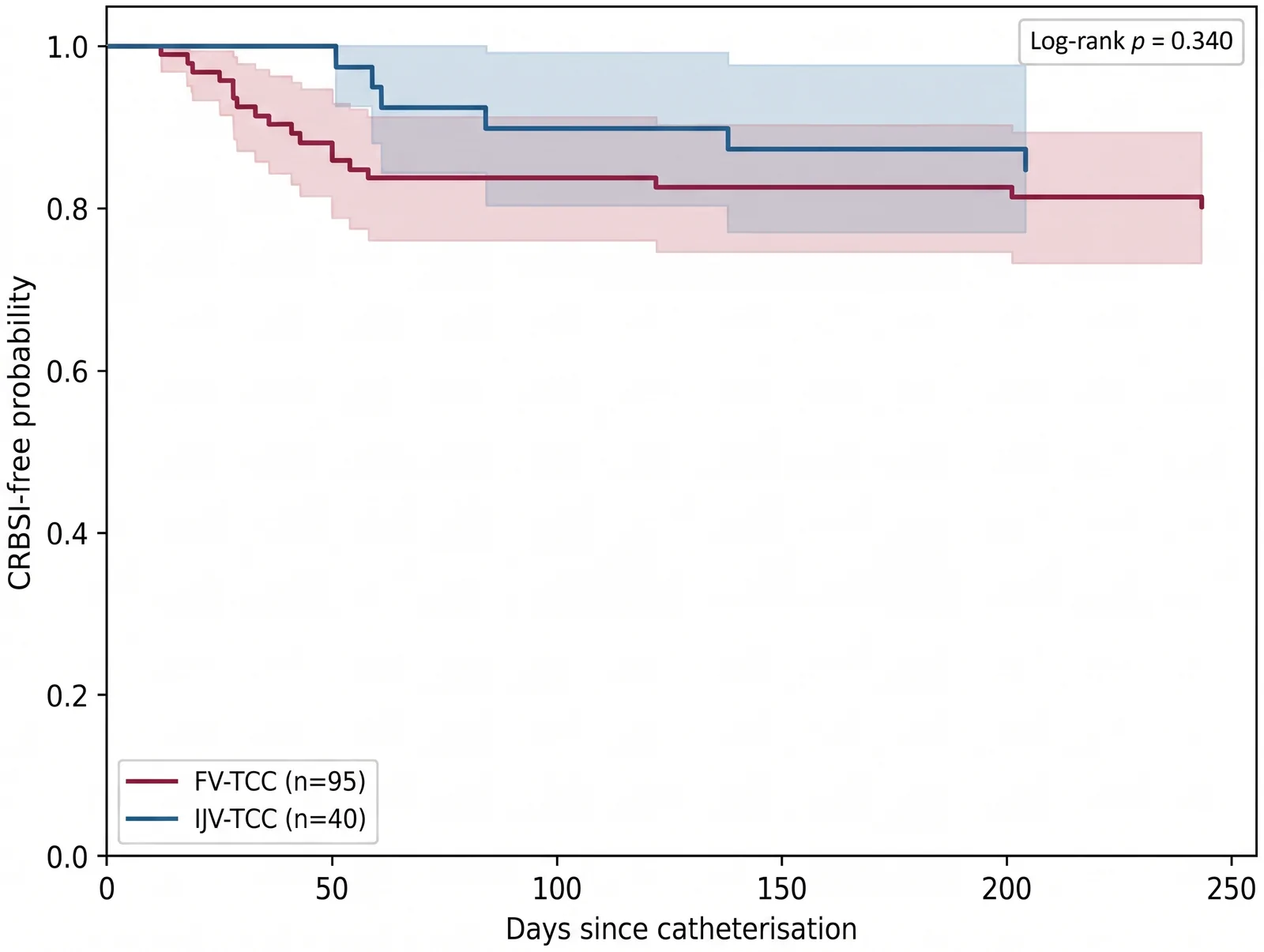
Kaplan–Meier CRBSI-free survival — index cohort (n = 135, first TCC per patient). FV-TCC n = 95, IJV-TCC n = 40. Event: first CRBSI; censored at catheter removal or end of follow-up. Log-rank FV vs IJV, p = 0.340 (non-significant).

**Fig 5:**
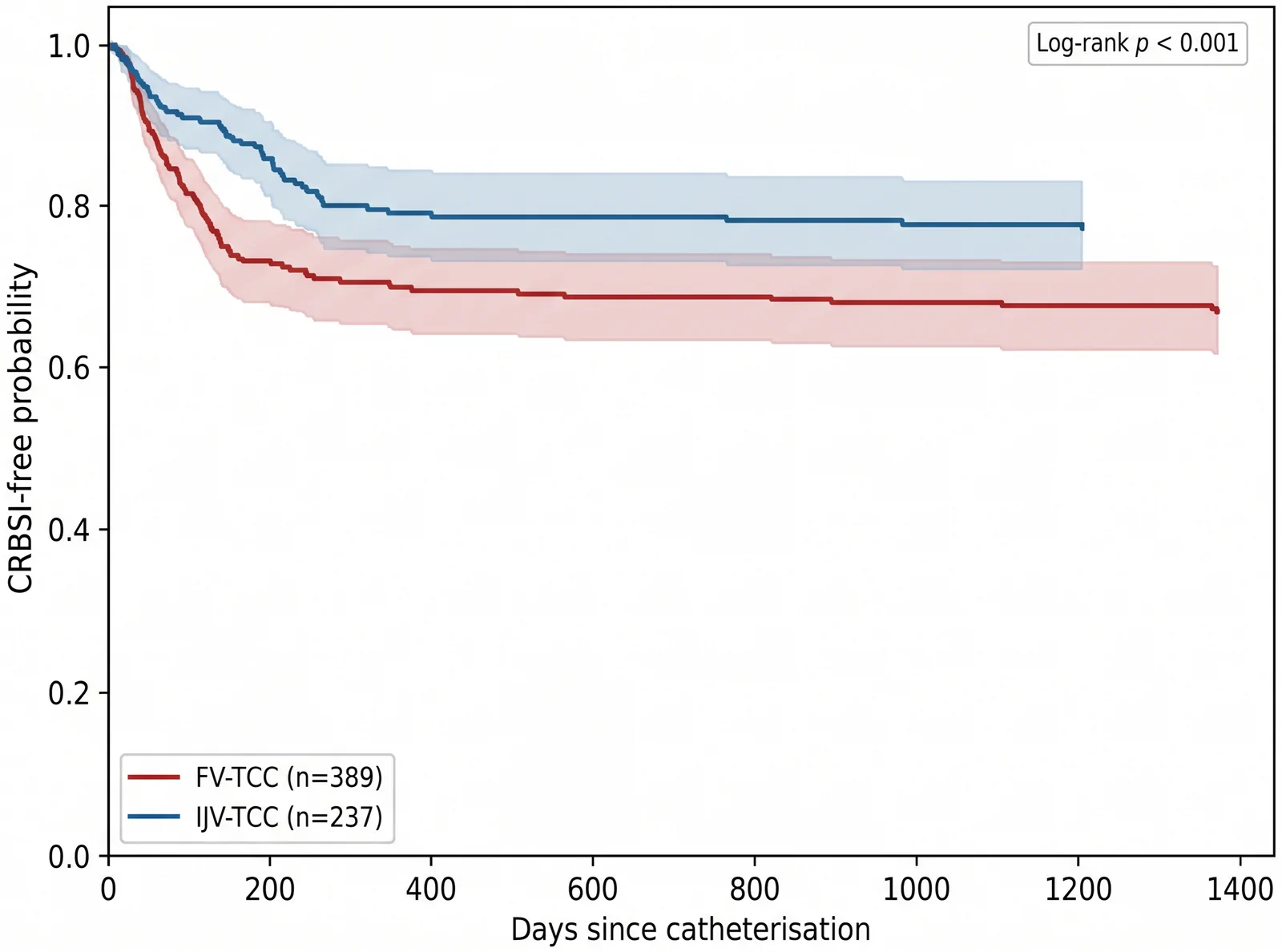
Kaplan–Meier CRBSI-free survival — all-TCC cohort (n = 626). FV-TCC n = 389, IJV-TCC n = 237. Log-rank p < 0.001 (unadjusted; see Cox model, Section 3.9, for adjusted estimate accounting for catheter sequence and patient clustering).

Among patients who developed CRBSI, the median time from catheter insertion to first infection was 34 days (IQR 20–58) in the index cohort and 56 days (IQR 27–133) in the all-TCC cohort. Among the 22 index-cohort patients whose first TCC was complicated by CRBSI, 18 (81.8%) subsequently required at least one additional catheter insertion; of these follow-on insertions, 38 were femoral and 11 were internal jugular. This proportion of femoral insertions (77.6%) was significantly higher than the expected 62.1% based on the overall registry femoral-to-IJV ratio (one-sample binomial test, p = 0.016), suggesting that prior CRBSI is associated with preferential subsequent routing to femoral access, consistent with progressive upper central venous exhaustion.

### 3.6. Catheter survival and dwell time

In the index cohort, median catheter dwell time was 51 days (IQR 27–115) for femoral vein TCCs (FV-TCC) and 68 days (IQR 28–202) for internal jugular vein TCCs (IJV-TCC). The most common reasons for catheter removal were maturation of an arteriovenous access (FV-TCC 26 [27.4%]; IJV-TCC 8 [20.0%]) and catheter-related bloodstream infection (CRBSI) (FV-TCC 16 [16.8%]; IJV-TCC 6 [15.0%]). Removal following AV access maturation was treated as a censoring event in time-to-event analyses, as it represents successful transition to permanent vascular access rather than catheter failure. Catheters remaining in-situ at end of follow-up or lost to follow-up (in-situ/LTFU) accounted for 26 (27.4%) of FV-TCC and 7 (17.5%) of IJV-TCC cases. Catheter blockage or poor flow was also a notable cause of removal, occurring in 8 (8.4%) of index FV-TCC and 4 (10.0%) of index IJV-TCC cases.

In the all-TCC cohort, late bleeding was more common in the IJV-TCC group than in FV-TCC (27 [11.4%] vs 25 [6.4%]; p = 0.036). As antiplatelet and anticoagulant use was similar between groups (44.3% vs 45.2%; p = 0.868), this is most likely attributable to the longer median dwell of IJV catheters (137 days vs 66 days for FV-TCC) rather than an intrinsic site hazard. Late bleeding events were predominantly low-grade and did not result in catheter removal in most cases.

Kaplan–Meier catheter survival curves are shown in Figs 6 and 7. The index cohort showed no statistically significant difference between sites (log-rank p = 0.950), though the small IJV group (n = 40) limits interpretive power. A significant difference was observed in the all-TCC cohort (p = 0.037), largely reflecting the shorter median dwell of femoral catheters (66 vs 137 days for IJV) and their concentration at higher sequence numbers.

**Fig 6:**
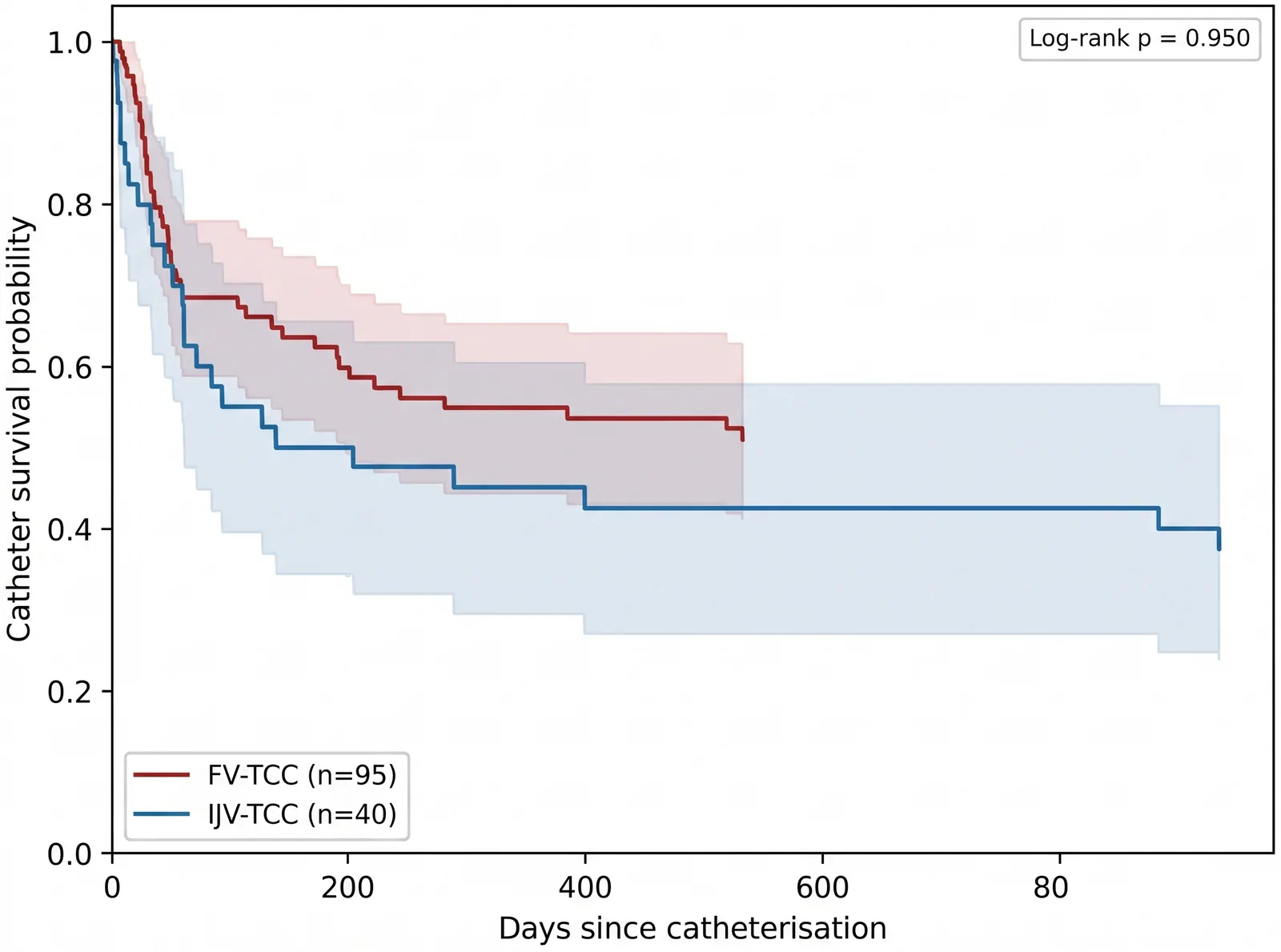
Kaplan–Meier catheter survival — index cohort (n = 135). Endpoint: catheter removal for any reason; AV maturation and in-situ at end of follow-up censored. Log-rank FV vs IJV, p = 0.950 (non-significant).

**Fig 7:**
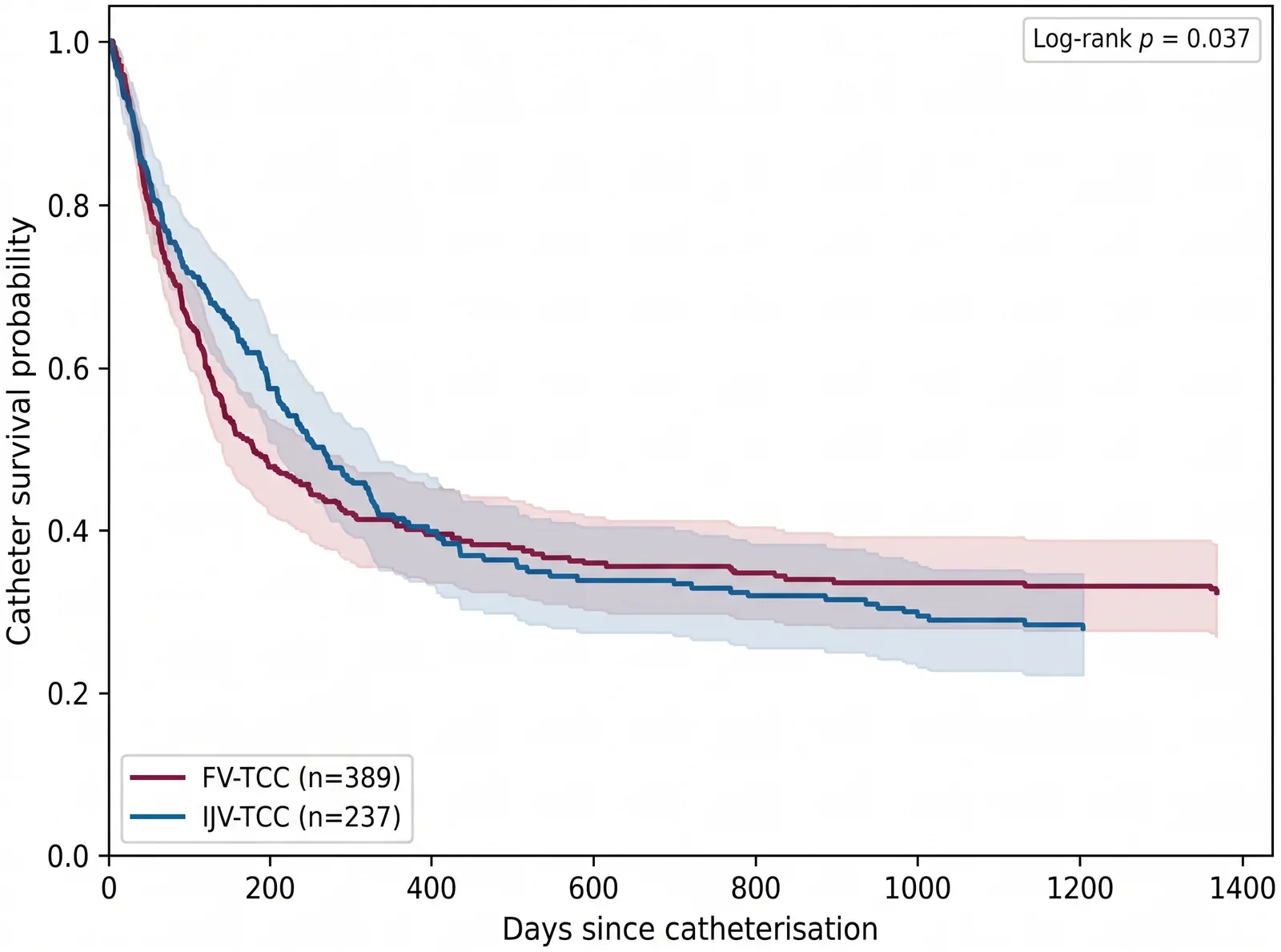
Kaplan–Meier catheter survival — all-TCC cohort (n = 626). Endpoint: catheter removal for any reason; AV maturation censored. Log-rank FV vs IJV, p = 0.037. Interpret with caution given within-patient clustering.

### 3.7. Patient survival

Fourteen patients (14.7%) in the femoral group and 11 (27.5%) in the jugular group died during follow-up (p = 0.093). This difference was not statistically significant; the numerical excess in the jugular group may reflect a higher baseline illness severity in patients routed to IJV access, though this was not formally assessed. Among deaths in the index cohort, the median time from catheter insertion to death was 76 days (IQR 23–385).

Kaplan–Meier patient survival curves were generated for both cohorts. Neither the index cohort (log-rank p = 0.487) nor the all-TCC cohort (log-rank p = 0.182) showed a statistically significant difference in patient survival by access site; the small event numbers in the index cohort mean that an effect cannot be formally excluded. No adjusted mortality model was fitted; the absence of a significant difference does not exclude a site effect on survival.

### 3.8. Within-patient clustering and sensitivity analyses

The all-TCC cohort comprised 626 catheter insertions from 339 unique patients. Among these, 145 patients (42.8%) underwent two or more catheter insertions during the study period, while 70 (20.6%) had three or more insertions; one patient contributed up to 12 catheters (Fig 8). Additionally, 47 patients (13.9%) had catheters inserted at both femoral and internal jugular sites at different time points, indicating that observations between access groups were not independent at the patient level.

**Fig 8:**
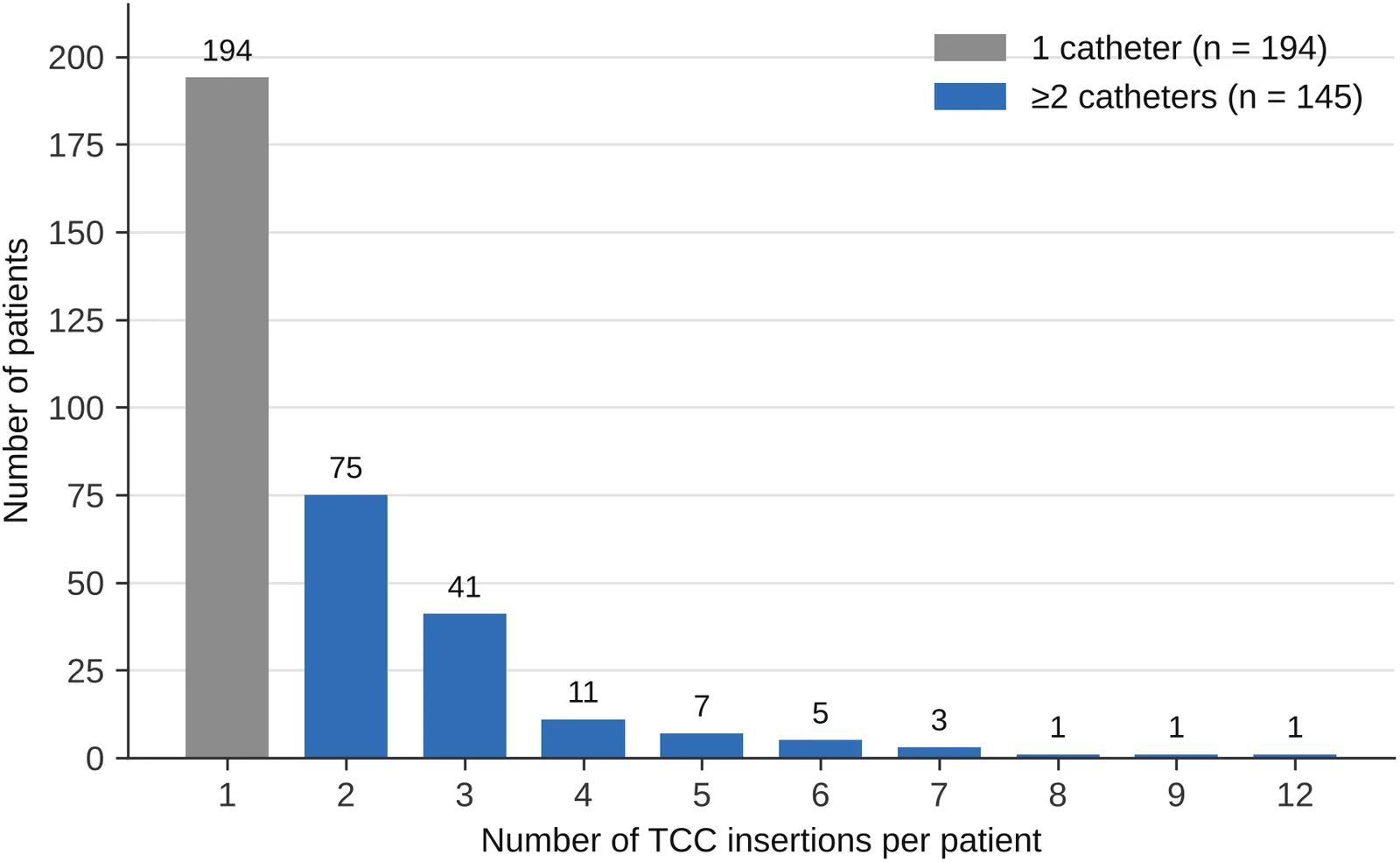
Distribution of catheter insertions per patient (All-TCC cohort, n = 339 patients). Frequency histogram showing the number of TCC insertions contributed by each patient over the study period. Most patients contributed one insertion (n = 194, 57.2%); 145 patients contributed two or more insertions (42.8%), providing the within-patient sequence data for the primary analysis.

Within-patient clustering was addressed in the multivariable Cox analysis (Section 3.9) using the marginal Lin–Wei–Ying approach with robust sandwich variance estimation, treating each patient as an independent cluster. The Kaplan–Meier and log-rank analyses presented in Sections 3.5–3.7 do not formally account for this clustering and should be interpreted descriptively; the adjusted Cox results provide the inferentially valid between-group comparison (Fig 9).

**Fig 9:**
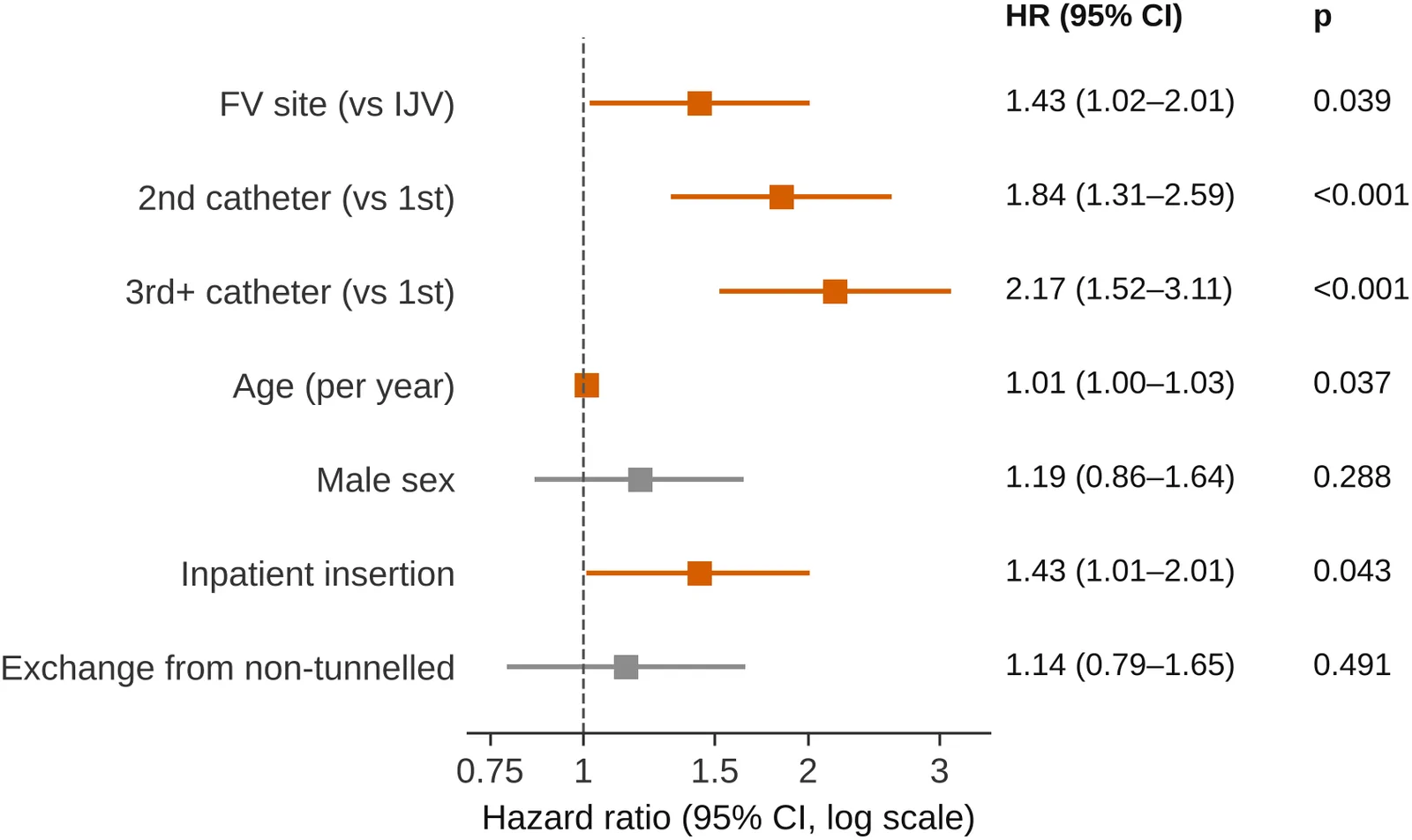
Forest plot of the cluster-robust multivariable Cox model (all-TCC cohort, n = 625; 154 CRBSI events). Catheter sequence number is the strongest independent predictor of CRBSI: second insertions (HR 1.84) and third-or-more insertions (HR 2.17) carry substantially greater hazard than first insertions. Femoral site (HR 1.43) remained associated with higher CRBSI hazard after full adjustment, alongside increasing age and inpatient insertion as additional independent predictors.

### 3.9. Multivariable analysis

A multivariable marginal Cox model with cluster-robust (sandwich) variance was fitted in the all-TCC cohort (n = 625; 154 CRBSI events). Catheter sequence was the strongest predictor: second insertions carried nearly twice the CRBSI hazard of first insertions (HR 1.84, 95% CI 1.31–2.59; p < 0.001) and third-or-more more than twice the hazard (HR 2.17, 95% CI 1.52–3.11; p < 0.001). After adjustment, femoral site remained significant but with an attenuated effect (HR 1.43, 95% CI 1.02–2.01; p = 0.039). Increasing age (HR 1.01 per year; p = 0.037) and inpatient insertion (HR 1.43; p = 0.043) were also independent predictors. The proportional hazards assumption was met for all covariates (global Schoenfeld test p = 0.31) (Table 4). The absolute CRBSI rate increment per sequence step (0.81/1000 catheter-days for 1st vs 2nd; 1.32/1000 for 1st vs 3rd+) exceeded the femoral site effect (0.95/1000 catheter-days with overlapping CIs), suggesting that sequence-related risk warrants at least as much clinical attention as site choice. In the ethnicity-adjusted sensitivity model, the femoral site HR was unchanged (HR 1.41, 95% CI 1.01–1.98; p = 0.045). When restricted to patients with three or fewer catheters (n = 310, 467 catheters), the escalating sequence pattern was preserved (1.04 vs 1.66 per 1000 catheter-days for first vs second insertions), indicating it is not driven by extreme outlier patients. In the pre-specified sensitivity analysis restricted to the 131 microbiologically confirmed CRBSI episodes (excluding the 23 clinically adjudicated probable cases), the sequence pattern remained intact (1st: 0.96; 2nd: 1.64; 3rd + : 2.35 per 1000 catheter-days), consistent with the primary analysis. In the index cohort (24 events total), catheter site was not statistically significant on univariable analysis (log-rank p = 0.340); given wide confidence intervals and only 24 events, this result reflects limited power rather than confirmed equivalence, and the index cohort analysis is therefore exploratory rather than inferential.

**Table 4:** Multivariable Cox model for CRBSI (all-TCC cohort).

Covariate	Hazard ratio	95% CI	p
FV site (vs IJV)	1.43	1.02–2.01	0.039
Age (per year)	1.01	1.00–1.03	0.037
Male sex	1.19	0.86–1.64	0.288
Inpatient insertion	1.43	1.01–2.01	0.043
Exchange from non-tunnelled	1.14	0.79–1.65	0.491
Catheter sequence: 2nd (vs 1st)	1.84	1.31–2.59	<0.001
Catheter sequence: 3rd+ (vs 1st)	2.17	1.52–3.11	<0.001

Marginal Cox proportional hazards model with Lin–Wei–Ying cluster-robust (sandwich) variance, treating each patient as a cluster to account for repeated catheter insertions. Model fitted on n = 625 catheter episodes from 339 patient clusters; 154 CRBSI events. Reference categories: IJV site, first catheter insertion, female sex, outpatient setting, no exchange. Time = days to first CRBSI per catheter episode; censored at catheter removal or end of follow-up. Proportional hazards assumption verified by scaled Schoenfeld residuals (global test p = 0.31; all individual covariates p > 0.05).

Because a subsequent catheter is often inserted after a preceding catheter fails (including failure due to CRBSI itself), the sequence-CRBSI association could partly reflect a subgroup of infection-prone patients rather than sequence number itself. To address this, we fitted an extended cluster-robust Cox model additionally adjusting for (i) previous CRBSI (any earlier catheter in the same patient complicated by CRBSI) and (ii) previous catheter removal specifically for CRBSI, alongside the original covariates. Catheter sequence remained an independent predictor of CRBSI after this adjustment (second insertion HR 1.81, 95% CI 1.13–2.91, p = 0.014; third-or-more HR 2.60, 95% CI 1.58–4.26, p < 0.001), while neither previous CRBSI (HR 0.72, 95% CI 0.37–1.40, p = 0.33) nor previous removal for CRBSI (HR 1.02, 95% CI 0.54–1.93, p = 0.95) was independently associated with subsequent CRBSI once sequence number, site, age, sex, inpatient setting and exchange were accounted for (Table 5). The persistence of the association after adjustment for the available prior-infection-history variables suggests that the sequence association is not fully explained by recurrently infected patients alone. However, residual confounding from incompletely captured prior access history, access-site trajectory, and removal indications remains possible. Data on previous access sites and the complete set of preceding catheter-removal reasons were not sufficiently complete or consistently coded to support a fully time-varying adjustment for all prior-access history; the two covariates included here (previous CRBSI and previous removal for CRBSI) represent the time-ordered variables that were reliably available in this dataset. Given this residual confounding and the observational design, catheter sequence number should be interpreted throughout as a marker of cumulative vascular-access complexity rather than a proven independent causal determinant.

**Table 5:** Extended multivariable Cox model for CRBSI, additionally adjusted for previous CRBSI and previous catheter removal for CRBSI (all-TCC cohort).

Covariate	Hazard ratio	95% CI	p
FV site (vs IJV)	1.42	0.98–2.07	0.067
2nd catheter (vs 1st)	1.81	1.13–2.91	0.014
3rd+ catheter (vs 1st)	2.60	1.58–4.26	<0.001
Age (per year)	1.01	1.00–1.02	0.041
Male sex	1.21	0.87–1.67	0.272
Inpatient insertion	1.38	0.97–1.97	0.077
Exchange from non-tunnelled	0.81	0.55–1.20	0.286
Previous CRBSI (any earlier catheter)	0.72	0.37–1.40	0.328
Previous catheter removed for CRBSI	1.02	0.54–1.93	0.952

Marginal Cox proportional hazards model with Lin–Wei–Ying cluster-robust (sandwich) variance, treating each patient as a cluster. Model fitted on n = 625 catheter episodes from 339 patient clusters; 154 CRBSI events. Reference categories: IJV site, first catheter insertion, female sex, outpatient setting, no exchange, no previous CRBSI, previous catheter not removed for CRBSI (or no previous catheter, for first insertions). This analysis was conducted for this revision in direct response to reviewer comments questioning whether the sequence-CRBSI association is confounded by prior infection history.

### 3.10. Temporal trend in CRBSI incidence

CRBSI incidence decreased over the study period from 2.15 per 1000 catheter-days in 2016 to 1.36 in 2021, with a transient elevation in 2020 (S2 Table and S1 Fig). The 2020 elevation temporally coincides with the COVID-19 pandemic; possible contributing factors, including disruptions to infection control staffing and deferral of elective vascular access procedures, cannot be assessed from this dataset, as contemporaneous procedural and staffing records were not captured. Whether any such factors influenced the observed increase at UMMC cannot be determined retrospectively. This limitation notwithstanding, the overall five-year downward trend is consistent with improving vascular access practice and is unlikely to be explained by the single-year 2020 fluctuation. This trend was not formally tested for statistical significance and should be considered descriptive only. Year of insertion was included as a covariate in the Cox model to assess whether catheters inserted in later years carried systematically different CRBSI risk; it was not independently associated with CRBSI after adjustment for sequence number and was not retained in the final model, suggesting the temporal decline does not materially confound the primary sequence findings.

### 3.11. Reason for catheter removal

In the all-TCC cohort, catheter removal due to catheter-related bloodstream infection (CRBSI) was more frequent in the femoral group compared with the internal jugular group (20.8% vs 15.6%). In contrast, removal due to arteriovenous fistula maturation was similar between groups (19.3% vs 16.5%) (S3 Table and S2 Fig).

## 4. Discussion

Principal findings. CRBSI incidence escalated with each successive catheter insertion (1.17, 1.98, and 2.49 per 1000 catheter-days for first, second, and third-or-more insertions). By the second catheter, CRBSI rates were numerically similar between FV and IJV access (31.7% each), indicating that insertion site is not the primary driver of infection risk in repeat-catheter patients. Clinically, the more relevant question shifts from “which site is safer?” to “which patients are at escalating risk across successive catheters?”

Catheter sequence number is a marker, not a direct cause: it reflects cumulative central venous instrumentation, access exhaustion, and patient complexity. Patients requiring a second or third TCC are more likely to carry subclinical biofilm burden from prior lines, progressive central venous stenosis, and greater comorbidity. Notably, 36.4% of those who developed CRBSI had a prior CRBSI on a previous catheter, suggesting an element of individual biological susceptibility independent of site or sequence. Chinese patients were more likely to have received jugular access (p = 0.033 index cohort, p = 0.014 all-TCC cohort); the reason is not clear from our data and may reflect referral patterns, AVF candidacy rates, or anatomical factors. A sensitivity model including ethnicity left the site HR essentially unchanged (HR 1.41), confirming that the ethnic imbalance did not confound the primary site-CRBSI association.

Comparison with prior literature. Restricting analysis to registry-index TCC insertions did not demonstrate a statistically significant difference between femoral and jugular access in CRBSI-free survival, catheter survival, or patient survival. With only 24 events and an unbalanced 70:30 FV-to-IJV ratio, this is best interpreted as an underpowered exploratory comparison rather than evidence of equivalence. Yaxley et al. [13], in 967 tunnelled catheter placements in 620 patients, found no significant difference in catheter survival by access site following multivariable adjustment (femoral HR 1.20, 95% CI 0.36–1.33), consistent with our index-cohort findings. Notably, Yaxley et al. did not adjust for catheter sequence number, which may have left residual confounding by patient complexity in their all-catheter estimate; the present study’s explicit sequence adjustment is the principal methodological advance over that work.

In the all-TCC cohort, after adjustment for catheter sequence number, age, sex, insertion setting, and exchange from a non-tunnelled catheter using a cluster-robust marginal Cox model, femoral access remained associated with a higher, though substantially attenuated, CRBSI hazard (HR 1.43, 95% CI 1.02–2.01; p = 0.039) compared with the unadjusted estimate. This attenuation is consistent with the sequence-stratified analysis: femoral access is concentrated at higher catheter sequence numbers, and by the second insertion FV and IJV carry similar CRBSI rates. The adjusted HR of 1.43, at borderline statistical significance, is best interpreted as a real but modest independent femoral site effect that is not fully explained by sequence confounding.

FV-TCC had shorter median dwell than IJV-TCC (51 vs 68 days in the index cohort; 66 vs 137 days in the all-TCC cohort). In the Cox model, time-at-risk is used as the time axis, so longer IJV dwell does not structurally disadvantage the IJV group; if anything, it increases the opportunity to observe IJV CRBSI, making the analysis conservative with respect to the observed femoral hazard. The shorter FV dwell likely reflects planned transition to permanent access rather than catheter failure, consistent with similar AV-maturation removal rates between sites. The higher rates of catheter blockage (20.7% vs 9.8%) and late bleeding (11.4% vs 6.4%) in IJV-TCC are attributable to the longer dwell time of jugular catheters rather than intrinsic site hazard, as discussed in Section 3.6. Exchange from a non-tunnelled catheter was retained in the Cox model regardless of statistical significance (HR 1.14, 95% CI 0.79–1.65; p = 0.491) on the basis of a priori biological plausibility: non-tunnelled catheters carry established biofilm colonisation risk, and any residual luminal biofilm present at the time of exchange may theoretically increase CRBSI susceptibility in the subsequently placed tunnelled catheter. Its inclusion did not alter the site or sequence HRs materially.

The higher proportion of inpatient FV-TCC insertions (61.7% vs 33.3% for IJV, p < 0.001) reflects that femoral access is preferentially used in acutely unwell patients with exhausted upper venous access. Even after adjusting for inpatient setting, unmeasured case-mix differences (acute illness severity, vascular exhaustion, nutritional depletion) in the femoral group persist and cannot be fully captured by a binary flag. Femoral site and inpatient setting are partially collinear, and their HRs (both 1.43) should be interpreted together. Pathogen profiles are reported in S1 Table. Gram-positive organisms predominated slightly in both access-site groups (FV: 47.6%, IJV: 39.2%), and gram-negative organisms were similarly represented (FV: 39.8%, IJV: 39.2%). The expected enrichment of enteric gram-negative organisms in femoral catheters due to proximity to the perineum was not evident in this dataset; however, the cohort was not powered to detect modest differences in pathogen distribution by site, and formal between-site microbiological comparisons were not pre-specified. This observation warrants further investigation in larger prospective studies.

Clinical implications. IJV access remains the first choice and should be used wherever feasible. When unavailable, the infection argument against femoral placement is more modest than previously suggested, particularly at first insertion (adjusted HR 1.43); however, DVT and limb oedema outcomes were not captured here and must be weighed independently, as prior data document ipsilateral DVT rates exceeding 10% for femoral TCCs [7]. For patients with future AVF plans, deliberate femoral use to preserve upper-limb vasculature remains a consideration when both infection and thrombotic risks have been assessed. For patients who may be candidates for kidney transplantation, femoral access should also be considered cautiously: femoral or iliac venous thrombosis or stenosis could complicate future allograft venous anastomosis or postoperative venous drainage. This outcome was not systematically assessed in the present cohort and is raised here as a clinical caveat rather than an observed study finding. Clinicians should monitor cumulative infection risk in patients undergoing a second or third catheter insertion, as risk rises with each reinsertion regardless of site.

Limitations and future directions. First, the retrospective single-center design limits causal inference; our center’s unusually high TCC prevalence, high femoral first-catheter rate (70%), and limited fluoroscopy may not reflect other practice settings. Second, selection bias is inherent: femoral access is reserved for more complex patients with failed or unavailable IJV access; unmeasured confounding by vascular status, nutritional status, and cumulative antibiotic exposure cannot be excluded. Third, the index cohort was underpowered for a formal site comparison: with only 24 CRBSI events, the null result (log-rank p = 0.340) cannot distinguish equivalence from insufficient power to detect the observed 2-fold numerical difference in incidence density (1.89 vs 0.94 per 1000 catheter-days). The clinical recommendation regarding femoral access therefore rests primarily on the adjusted all-TCC Cox model. Fourth, DVT and limb oedema were not systematically screened for (no protocol-driven duplex ultrasonography), and long-term functional outcomes were not captured; however, catheter removal specifically attributed to DVT was rare in this cohort (1 of 626 catheters, 0.16%; a femoral TCC), indicating that clinically apparent DVT requiring device removal was uncommon, although subclinical or non-removal-associated DVT cannot be excluded and should be assessed systematically in future prospective work. Fifth, 23 of 154 CRBSI events (14.9%) were clinically adjudicated probable CRBSI without microbiological confirmation; these were included per IDSA criteria with systematic adjudication, and a pre-specified sensitivity analysis restricted to the 131 microbiologically confirmed events yielded identical patterns.

## 5. Conclusion

The SEQUEL Study examined 626 tunnelled-cuffed catheter insertions in 339 patients over five years. CRBSI incidence rose with each successive catheterisation, from 1.17 per 1000 catheter-days at first insertion to 1.98 at second and 2.49 at third or beyond; by the second catheter, femoral and jugular access carried similar infection rates. In the fully adjusted cluster-robust Cox model, catheter sequence was a stronger predictor of CRBSI than insertion site. The femoral site effect (HR 1.43) was substantially attenuated after sequence adjustment, and the registry-index cohort analysis, while consistent with this direction, was underpowered to be inferential. When jugular access is not feasible, femoral TCC placement may be an acceptable alternative from an infection-risk perspective, but DVT and limb complications must be independently evaluated before this conclusion is applied clinically. These findings support closer surveillance and targeted infection-prevention strategies for patients requiring repeated TCC insertions, as catheter sequence number is a stronger and more actionable risk marker than insertion site alone.

## Supporting information

S1 Checklist STROBE Statement checklist of items to be included in reports of observational studies, completed for the SEQUEL Study. (DOCX)

S1 Table CRBSI microbiology and clinical outcomes, by cohort and access site. (DOCX)

S2 Table CRBSI incidence by year of insertion (all-TCC cohort), 2016–2021. (DOCX)

S3 Table Reason for catheter removal by site and cohort. (DOCX)

S1 Fig Annual TCC insertions and CRBSI incidence rate, 2016–2021. Bars (left axis) = number of insertions per year; line (right axis) = CRBSI rate per 1000 catheter-days with 95% Poisson confidence interval shading. The shaded 2020 column marks the COVID-19 pandemic year, which showed a transient rise in CRBSI incidence.
(TIF)

S2 Fig Reason for catheter removal by access site (all-TCC cohort, n = 626). CRBSI accounted for 20.8% of FV-TCC removals vs 15.6% of IJV-TCC removals. AV maturation rates were similar (19.3% vs 16.5%), indicating that the higher FV removal rate reflects infection burden rather than elective transition to permanent access. cd, catheter-days; AV, arteriovenous; LTFU, lost to follow-up.
(TIF)

## Abbreviations

AV: arteriovenous
AVF: arteriovenous fistula
BCF: brachiocephalic fistula
CI: confidence interval
CRBSI: catheter-related bloodstream infection
DM: diabetes mellitus
DVT: deep vein thrombosis
ESRD: end-stage renal disease
FV: femoral vein
HR: hazard ratio
HTN: hypertension
IDSA: Infectious Diseases Society of America
IJV: internal jugular vein
INR: international normalised ratio
IQR: interquartile range
KDOQI: Kidney Disease Outcomes Quality Initiative
LTFU: lost to follow-up
MDTR: Malaysian Dialysis and Transplant Registry
MREC: Medical Research Ethics Committee
NTDC: non-tunnelled dialysis catheter
STROBE: Strengthening the Reporting of Observational Studies in Epidemiology
TCC: tunnelled-cuffed catheter
UMMC: Universiti Malaya Medical Centre
