# Supplementary material for: SEQuential tUnneled Catheters and Infective Events in haemodiaLysis: A Five-Year Retrospective Cohort Study (SEQUEL Study)
Source: PLoS One. 2026 Nov 4;21(11):e0362091. doi: 10.1371/journal.pone.0362091 (PMC13734602; doi:10.1371/journal.pone.0362091)
Supplement: Supplementary file 1 [file pone.0362091.s008.pdf]

Universiti Malaya,
Department of Medicine,
Faculty of Medicine,
Kuala Lumpur, Malaysia.

PLOS ONE
Public Library of Science

Response to the Academic Editor and Reviewers — SEQUEL Study

Manuscript: "SEQuential tUnneled Catheters and Infective Events in haemodiaLysis: A Five-Year Retrospective Cohort Study (SEQUEL Study)"

Thank you for the opportunity to revise our manuscript. We thank the Academic Editor and all four reviewers for their careful and constructive evaluation. We have revised the manuscript to address every point raised, and respond point-by-point below, with section references to the revised manuscript. A clean revised manuscript, an updated STROBE checklist, individually filed figures, and Supporting Information files accompany this letter; a page-and-section summary of the corrections made in this round is provided in the table near the end of this letter.

Journal (Editorial) Requirements

Comment 1:

Please ensure that your manuscript meets PLOS ONE's style requirements, including those for file naming.

Response: The manuscript has been reformatted to PLOS ONE style: the title page (bold title, superscript-numbered authors, numbered affiliations, named corresponding author with e-mail) and body follow the PLOS ONE sample templates. Figures are no longer embedded in the manuscript file; each is supplied as an individual, simply-named file (Fig1.jpg – Fig9.png) for separate upload, and Supporting Information files are named per PLOS convention (S1 Checklist, S1–S3 Table, S1–S2 Fig). The manuscript file itself has been renamed without spaces (SEQUEL_Study_Revised_Manuscript.docx). [Manuscript: p.1, title page; file naming applies to the accompanying figure/SI files, not manuscript body text]

Comment 2:

Your ethics statement should only appear in the Methods section of your manuscript.

Response: The ethics approval and consent statement previously appeared only in the end-of-manuscript Declarations section, not in Methods. We have moved it verbatim to the start of the Materials and Methods section (Section 2.2, "Ethics statement") and removed it from Declarations, so it now appears exactly once, in Methods. [Manuscript: p.4, lines 14–15]

Comment 3:

Please explain your reasons for restricting data availability, in accordance with PLOS data-sharing policy.

Response: We have revised the Data Availability Statement in Section 6.0 (Declarations) to state our restriction more concretely. The minimal dataset contains potentially re-identifiable patient-level clinical data and cannot be publicly deposited because public disclosure would be inconsistent with the approval granted by the Medical Research Ethics Committee, Universiti Malaya Medical Centre (MREC No. 20211117-10778), and applicable Malaysian patient-data privacy requirements. De-identified data sufficient to reproduce the reported aggregate findings, together with the statistical analysis code, may be requested from the corresponding author at ccgan@ummc.edu.my; access is subject to institutional data-governance review and confirmation of appropriate ethics approval, where required. We have entered the same statement in the online submission system's Data Availability field and understand this exemption request will be escalated for editorial approval. [Manuscript: p.21, line 32]

Comment 4:

Please amend either the abstract on the online submission form or the abstract in the manuscript so that they are identical.

Response: The manuscript abstract is now the authoritative, final version (it incorporates the corrected log-rank p-value described under Reviewer 2, comment 5, below, and the IJV terminology correction described in the corrections table). We ask the Editorial Office / corresponding author to paste this exact text into the "Edit Submission" abstract field, as we do not have direct access to the online submission form from this revision workflow. The final abstract text is reproduced in full at the end of this letter for ease of copying. [Manuscript: p.1–2, Abstract]

Comment 5:

Please ensure that you refer to Figure 1 and Figure 2 in your text.

Response: Explicit in-text citations "(Figure 1)" and "(Figure 2)" have been added at the end of the sentences introducing the cohort flow (Section 3.1) and the sequence-stratified CRBSI incidence (Section 3.4), respectively. [Manuscript: p.7, line 33 (Figure 1); p.11, lines 8–9 (Figure 2)]

Comment 6:
We notice that your supplementary figures/tables are included in the manuscript file. Please remove them and upload them with the file type 'Supporting Information'.

Response: Supplementary Table 3, Supplementary Table 5, Supplementary Table 6, Supplementary Figure 10 and Supplementary Figure 11 have been removed from the manuscript file and are now supplied as separate Supporting Information files (S1–S3 Table, S1–S2 Fig). Their legends are listed in a "Supporting Information" section placed after the reference list, as requested. In extracting this material we also noticed that the narrative text describing it (Sections 3.10 "Temporal Trend in CRBSI Incidence" and 3.11 "Reason for Catheter Removal") had been misplaced after the References/Declarations in the originally submitted file; we relocated this Results narrative back into the Results section, immediately after Section 3.9, where it belongs. [Manuscript: p.17, line 17 (Section 3.10); Supporting Information, p.24, line 1]

Comment 7:
Please include a copy of Table 3 which you refer to in your text on page 11.

Response: Table 3 (CRBSI incidence density by cohort and access site, with exact-Poisson 95% confidence intervals) was indeed missing from the originally submitted file, although the surrounding text already cited it and reported its values. We independently recomputed these incidence densities from the underlying study dataset; the recomputed values match those already stated in the text exactly (Index FV 1.89 [1.12–2.99]; Index IJV 0.94 [0.34–2.04]; All-TCC FV 2.08 [1.70–2.52]; All-TCC IJV 1.04 [0.77–1.36] per 1000 catheter-days), and the table has been inserted at its citation point in Section 3.5. To avoid duplicating this information, we have also removed the CRBSI-incidence-density row from Table 1, which now reports baseline, procedural and outcome characteristics only, with a footnote directing readers to Table 3 for incidence density. [Manuscript: p.12, line 24]

Comment 8:
Reviewer-recommended citations.

Response: No reviewer explicitly recommended specific additional publications to cite in this round; accordingly, no new citations have been added on this basis. [Not applicable — no manuscript location]

Reviewer 1

Overall assessment: technically sound; statistics appropriate; clearly written; minor revisions requested to improve methodological transparency, statistical clarity, and balanced interpretation.

Comment 1:

Expand methodological detail on sequence assignment, CRBSI adjudication, and handling of missing data.

Response: We have expanded Section 2.4 (Definitions) to provide an explicit, operational definition of clinically adjudicated probable CRBSI: compatible systemic features (fever, rigors, or haemodynamic instability) in a patient with a TCC; no alternative infectious source identified after clinical assessment and available microbiological investigations; and clinical improvement following catheter-directed management and/or antimicrobial therapy, with cases having a documented non-catheter source of infection excluded. We also clarify the approach to culture-negative presentations and acknowledge that pre-culture antibiotic exposure was not consistently available in this retrospective dataset. Section 2.6 (Statistical Analysis) states the missing-data handling (one record missing CRBSI outcome excluded from event-based analyses; three records with missing INR excluded from INR comparisons only; no imputation). Catheter sequence numbering (chronological, per patient, within the registry) is defined in Sections 2.4, 2.5.1, and 2.6; we have not altered this description as reviewers did not identify a specific ambiguity. We believe these revisions clarify the sequence-assignment approach and its limitations. [Manuscript: p.4, line 36 (Section 2.4); p.6, line 13 (Section 2.6)]

Comment 2:

Present sensitivity analyses more prominently and integrate them into the main results narrative.

Response: Section 3.9 already reports three pre-specified sensitivity analyses (ethnicity-adjusted model; restriction to ≤3 catheters; restriction to microbiologically confirmed CRBSI). In direct response to Reviewer 2 (below) we have added a fourth, new sensitivity analysis (Table 5) adjusting for previous CRBSI and previous catheter removal for CRBSI, presented immediately after the primary Cox model and forest plot for visibility. [Manuscript: p.15, line 28 – p.17 (Section 3.9, Table 5)]

Comment 3:

Provide confidence intervals and more detailed breakdowns of incidence rates.

Response: The newly inserted Table 3 supplies exact-Poisson 95% confidence intervals for all four cohort×site incidence-density estimates cited in the text, addressing this point directly (see A.7 above). [Manuscript: p.12, line 24 (Table 3)]

Comment 4:

Strengthen the discussion by balancing the sequence versus site findings and elaborating on clinical implications.

Response: We have added a new paragraph to Section 3.9 (and Table 5) demonstrating that the sequence effect persists after adjustment for prior infection history, and strengthened the quantification of femoral access as predominantly rescue rather than planned (Section 3.3), both of which sharpen the sequence-versus-site framing already present in the Discussion. We have also added an explicit transplantation-candidacy caveat to the Clinical Implications paragraph (Section 4.0; see Reviewer 4, comment 2, below). [Manuscript: p.10, lines 23–24 (Section 3.3); p.20, lines 9–18 (Section 4.0)]

Comment 5:

Revise for clarity, consistency, and conciseness, correcting minor language issues.

Response: We corrected an internal numerical inconsistency (the index-cohort log-rank p-value was reported as 0.435 in the text/abstract but 0.340 in Figure 4 — see Reviewer 2, comment 5), corrected three residual "IJ" abbreviations to "IJV" for consistency, standardised "single-centre" to "single-center" throughout (PLOS ONE house style), and reviewed the manuscript for consistency of terminology (see Reviewer 2, comment 1). [Manuscript: p.2, line 1; p.13, line 4; p.15, line 36; p.20, line 29 (log-rank p = 0.340, all instances)]

Comment 6:

Expand the limitations section to acknowledge residual confounding and generalisability concerns.

Response: The Limitations paragraph already acknowledges residual confounding (unmeasured vascular, nutritional, and antibiotic-exposure factors) and generalisability (single-center design,

unusually high TCC prevalence, limited fluoroscopy). We have additionally corrected and quantified the DVT-related limitation and added a kidney-transplant venous-access caveat (see Reviewer 3 and Reviewer 4 below), and quantified the femoral rescue-access selection bias (Reviewer 2, comment 3), which further strengthens this section. [Manuscript: p.20, line 23]

Reviewer 2

Comment 1:

The catheter sequence number is not necessarily the patient's true catheter sequence... The terminology should be changed from 'first-ever TCC' to 'registry-index TCC'.

Response: We agree, and in fact the submitted manuscript already used "registry-index cohort/TCC" as its primary terminology throughout the Abstract, Methods, and Results (the literal phrase "first-ever TCC" does not appear in the manuscript). For full consistency we changed the one remaining bare reference ("index TCC insertions" in the Discussion, Section 4.0 "Comparison with prior literature") to "registry-index TCC insertions", and confirmed that every other mention of "index cohort/TCC" is explicitly defined, at first use in each section, as the registry-recorded first TCC rather than a verified lifetime-first catheter. [Manuscript: p.18, line 33]

Comment 2:

The sequence effect may not be caused by sequence itself... The authors should adjust for previous CRBSI, preceding catheter-removal reasons, and previous sites. Until then, catheter sequence should be interpreted as a risk marker and not an independent causal determinant.

Response: We agree this is an important test of the sequence hypothesis and have performed it. We fitted a new extended cluster-robust Cox model (Table 5) adding two time-ordered, patient-history covariates that were available reliably in the dataset: (i) previous CRBSI (whether any earlier catheter in the same patient had already been complicated by CRBSI) and (ii) previous catheter removal specifically for CRBSI. After adjustment for these measured prior-infection-history variables, higher registry catheter sequence remained independently associated with subsequent CRBSI hazard (2nd catheter HR 1.81, 95% CI 1.13–2.91, $p = 0.014$; 3rd+ HR 2.60, 95% CI 1.58–4.26, $p < 0.001$), while neither previous CRBSI (HR 0.72, $p = 0.33$) nor previous removal for CRBSI (HR 1.02, $p = 0.95$) was itself independently associated with subsequent CRBSI. Complete prior-access-site history and all preceding catheter-removal reasons were not sufficiently complete or consistently coded in this retrospective dataset to support a fully time-varying adjustment; we have acknowledged this as a residual-confounding limitation in Section 3.9 and the Limitations paragraph.

Given this residual confounding and the observational design, we now describe catheter sequence, throughout the manuscript, as a marker of cumulative vascular-access complexity rather than a proven independent causal determinant, per the reviewer's recommendation. [Manuscript: p.16, line 26 (Section 3.9); p.17, line 9 (Table 5)]

Comment 3:
There is a confounding issue regarding the femoral vs jugular comparison... the study compares jugular access with a predominantly rescue-access femoral group.

Response: We agree and have quantified this directly from the procedural-indication data. Of 389 femoral insertions in the all-TCC cohort, 359 (92.3%) followed absent/unsuitable IJV access or a failed IJV attempt at the same sitting; only 2 (0.5%) followed patient preference with no antecedent IJV issue, and in the registry-index cohort specifically only 1 of 95 (1.1%) femoral catheters represented a primarily planned femoral approach. We have added this quantification to Section 3.3, and it now explicitly frames the femoral group as predominantly rescue-access, consistent with the reviewer's reading; the existing Limitations paragraph already names this selection mechanism, and we have left that language in place alongside the new numbers. [Manuscript: p.10, lines 23–24 (Section 3.3); p.12, line 11 (Section 3.5)]

Comment 4:
The definition of 'clinically adjudicated probable CRBSI' needs further explanation... criteria for culture-negative cases are insufficiently described.

Response: We have expanded Section 2.4 (Definitions) to give an operational definition of clinically adjudicated probable CRBSI: compatible systemic features, no clinically identified alternative infection source after available investigations, and clinician adjudication based on IDSA-aligned principles, with clinical improvement following catheter-directed management and/or antimicrobial therapy. We also clarify that culture-negative status was interpreted in the context of documented pre-culture antimicrobial exposure and acknowledge that this exposure was not consistently recorded in our retrospective dataset. Twenty-three of 154 events (14.9%) met this definition, and a pre-specified sensitivity analysis restricted to the 131 microbiologically confirmed events (Section 3.9) shows an unchanged pattern, which we independently reproduced from the dataset. We have removed the earlier reference to a "pre-defined IDSA criteria checklist": adjudication followed the operational definition above and IDSA clinical principles for probable catheter-related bacteraemia (reference 8), applied by the attending nephrologist with independent

review by the lead investigator, rather than a separately archived scoring instrument. [Manuscript: p.4, line 36 (Section 2.4)]

Comment 5:

Inconsistencies were noted in the figures. The manuscript reports index-cohort log-rank p-value of 0.435, but figure 4 mentions p value of 0.340.

Response: We independently recomputed the index-cohort (FV vs IJV) CRBSI-free log-rank test directly from the underlying dataset (18/95 FV and 6/40 IJV CRBSI events). The recomputed p-value is 0.340, matching Figure 4 exactly. The text and abstract value of "0.435" was therefore the error and has been corrected to 0.340 in all five locations where it appeared (Abstract, Results Section 3.5, Section 3.9, and the Limitations paragraph), so the manuscript is now internally consistent with Figure 4. [Manuscript: p.2, line 1; p.13, line 4; p.15, line 36; p.20, line 29]

Comment 6:

Figure 8 shows the bars with 194 patients with one catheter and 145 with two or more, which is consistent with the text, but the legend says n=238 and n=101.

Response: Confirmed and corrected. We independently reconstructed the per-patient catheter-count distribution from the dataset (194 patients with 1 catheter; 75 with 2; 41 with 3; 11 with 4; 7 with 5; 5 with 6; 3 with 7; and 1 each with 8, 9 and 12), which sums to 145 patients with ≥2 catheters and 339 patients overall — matching the bars and the in-text caption exactly. Only the in-chart legend box (which read "n=238"/"n=101") was in error; Figure 8 has been regenerated with the corrected legend ("1 catheter (non-clustered, n=194)"; "≥2 catheters (clustered, n=145)"). [Manuscript: p.15, line 11]

Reviewer 3

Comment 1:

The study is statistics heavy. Simpler language may help.

Response: We have reviewed the manuscript for opportunities to simplify statistical language without losing precision and have kept new additions (Tables 3 and 5) in plain HR/CI/p format consistent with the rest of the manuscript. [Manuscript: p.12, line 24 (Table 3); p.17, line 9 (Table 5)]

Comment 2:

This is a femoral vein-centric approach, which is not routinely performed in other centres. It has been deployed for failed IJV insertion even in first timers — seems unusual.

Response: This reflects local practice patterns at our centre, discussed in the Limitations paragraph (unusually high TCC prevalence and high femoral first-catheter rate). The quantification in Section 3.3 (92.3% of femoral insertions following absent/failed IJV access) clarifies that femoral use, including at first insertion, is overwhelmingly reactive to IJV unavailability or failure rather than a primary practice preference. [Manuscript: p.20, line 23]

Comment 3:

What was the exact IDSA criteria used for probable CRBSI?

Response: See response to Reviewer 2, comment 4, above. Section 2.4 now gives an explicit, operational definition of clinically adjudicated probable CRBSI and cites IDSA clinical principles for probable catheter-related bacteraemia (reference 8), together with the systematic clinician-adjudication process used when microbiological confirmation was unavailable. [Manuscript: p.4, line 36 (Section 2.4)]

Comment 4:

Any particular reason why femoral catheters did not have GNB as expected?

Response: This is addressed in the Discussion (Section 4.0): gram-negative organisms were similarly represented at both sites (FV 39.8% vs IJV 39.2%), and we note the cohort was not powered for a pre-specified between-site microbiological comparison, so this null finding should be interpreted cautiously and warrants larger prospective study, as stated in the text. [Manuscript: p.20, lines 2–3]

Comment 5:

Suggestion of femoral catheters as an alternative should be with a caveat: DVT has not been addressed.

Response: We have revised the Discussion and Limitations to state this more precisely. DVT and limb oedema were not systematically screened for (no protocol-driven duplex ultrasonography), but we checked the dataset for catheter removals attributed to DVT: only 1 of 626 catheters (0.16%, a femoral TCC) was removed for this reason, indicating that clinically apparent DVT requiring device removal was uncommon in this cohort, although subclinical or non-removal-associated DVT cannot

be excluded absent systematic screening. The Clinical Implications paragraph (Section 4.0) already cites published ipsilateral femoral DVT rates exceeding 10% (reference 7) as an explicit caveat on any recommendation favouring femoral access, and now additionally states that femoral or iliac venous thrombosis or stenosis could complicate future kidney-transplant allograft venous anastomosis or postoperative venous drainage in patients who may be transplant candidates; this was not systematically assessed in our retrospective cohort and is presented as a clinical caveat, not a study finding (see Reviewer 4, comment 2, below). [Manuscript: p.2, line 18 (Abstract); p.20, line 33 (Limitations)]

Reviewer 4

Comment 1:

Because you used so many abbreviations, make a list for them.

Response: An Abbreviations list has been added to the Declarations section (Section 6.0), immediately after the Data Availability Statement. [Manuscript: p.22, line 6]

Comment 2:

Femoral vein cannulation is associated with increasing risk of DVT including iliac veins, and if there is a future plan for kidney transplantation, it may complicate the procedure.

Response: We have expanded the Discussion and Limitations to state directly that femoral or iliac venous thrombosis or stenosis may be particularly consequential in patients being considered for kidney transplantation, because it could complicate future allograft venous outflow. This outcome was not systematically screened for in this retrospective cohort; our findings should therefore not be interpreted as establishing femoral TCC safety with respect to thrombotic or transplant-related outcomes. The Clinical Implications paragraph continues to caution that thrombotic and limb complications must be weighed independently before recommending femoral access, and that femoral use to preserve upper-limb vasculature for future AVF creation is only a consideration once both infection and thrombotic risk have been assessed. [Manuscript: p.20, lines 16–18]

Corrections made:

The table below summarises the specific corrections made in this revision, with page references to the accompanying revised manuscript (Word pagination; final PLOS ONE typesetting may repaginate).

Page	Section	Correction
p.1	Title page	Added the corresponding author's name (Chye Chung Gan) alongside the e-mail address, per PLOS ONE title-page requirements.
p.1	Abstract, Results	Corrected "237 IJ" to "237 IJV" for consistent abbreviation use.
p.3	Section 1.0, Introduction	"single-centre" corrected to "single-center" (PLOS ONE / US English house style).
p.4	Section 2.3, Catheter insertion protocol	"IJ insertion" corrected to "IJV insertion" for consistent abbreviation use.
p.4–5	Section 2.4, Definitions	Added an explicit, operational definition of clinically adjudicated probable CRBSI (compatible features, exclusion of alternative source, clinical improvement, treatment of culture-negative status); removed the unsupported reference to a "pre-defined IDSA criteria checklist"; added acknowledgement that pre-culture antibiotic exposure was not systematically recorded.
p.5	Section 2.5.1, Inclusion and Exclusion Criteria	Formatting: removed residual left-indentation (blockquote-style spacing) left over from document conversion.
p.8	Table 1	Removed the CRBSI-incidence-density row (duplicated Table 3); Table 1 now reports baseline, procedural and outcome characteristics only.
p.10	Table 1 footnote	Added a cross-reference directing readers to Table 3 for CRBSI incidence density with 95% confidence intervals.
p.11	Table 2 and Section 3.3 footnote	"Failed IJ TCC attempt" corrected to "Failed IJV TCC attempt" (table cell and footnote).
p.15	Section 3.8	"(figure 8)" corrected to "(Figure 8)" for consistent capitalisation.
p.16–17	Table 5	Denominator corrected from n = 626 to n = 625 catheter episodes, matching the primary Cox model (one record with missing CRBSI outcome excluded from all event-based analyses).
p.17	Section 3.9, Multivariable Analysis	Expanded the extended-model narrative to state that prior-site and complete removal-reason data were not fully available for time-varying adjustment, and to describe catheter sequence as a

		marker of cumulative access complexity rather than a proven causal determinant.
p.20	Section 4.0, Discussion — Clinical implications	Added an explicit caveat that femoral/iliac venous thrombosis or stenosis could complicate future transplant allograft venous anastomosis, framed as a clinical caveat rather than an observed outcome.
p.22	Section 6.0, Data Availability Statement	Revised wording to state more concretely how, and to whom, de-identified data and analysis code may be requested.
p.23	References	Normalised paragraph/style formatting of reference-list entries 9–14 for visual consistency with entries 1–6.

We are grateful to the Academic Editor and reviewers for their careful and constructive review, which has materially strengthened the manuscript. We believe the revised manuscript addresses all points raised and hope it is now suitable for publication in PLOS ONE.

Yours sincerely,

Corresponding Author:
Chye Chung Gan
Nephrology Unit, Department of Medicine
Faculty of Medicine, Universiti Malaya Medical Centre
Email: ccgan@ummc.edu.my
