# Supplementary material for: SEQuential tUnneled Catheters and Infective Events in haemodiaLysis: A Five-Year Retrospective Cohort Study (SEQUEL Study)
Source: PLoS One. 2026 Nov 4;21(11):e0362091. doi: 10.1371/journal.pone.0362091 (PMC13734602; doi:10.1371/journal.pone.0362091)
Supplement: Supplementary file 2 [file pone.0362091.s001.docx]

**STROBE Checklist — SEQUEL Study**

Sequential tunnelled catheters and infective events in haemodialysis: a five-year retrospective cohort study

Study design: Retrospective cohort study | Journal: PLOS ONE | MREC No. 20211117-10778

| **STROBE Statement — Checklist of items that should be included in reports of cohort studies** | | | |
| --- | --- | --- | --- |
| **Item** | **Recommendation** | **Reported** | **Location in manuscript** |
| **Title and Abstract** | | | |
| 1(a) | Indicate the study's design with a commonly used term in the title or abstract | **Yes** | Title; Abstract (Methods) |
| 1(b) | Provide in the abstract an informative and balanced summary of what was done and what was found | **Yes** | Abstract |
| **Introduction** | | | |
| 2 | Explain the scientific background and rationale for the investigation being reported | **Yes** | Introduction, paragraphs 1–3 |
| 3 | State specific objectives, including any prespecified hypotheses | **Yes** | Introduction, paragraph 4 |
| **Methods** | | | |
| 4 | Present key elements of study design early in the paper | **Yes** | Section 2.1 (Study design) |
| 5 | Describe the setting, locations, and relevant dates, including periods of recruitment, exposure, follow-up, and data collection | **Yes** | Sections 2.1, 2.3; data accessed 31/03/2022 |
| 6(a) | Cohort study — Give the eligibility criteria, and the sources and methods of selection of participants. Describe methods of follow-up | **Yes** | Sections 2.1, 2.4 |
| 6(b) | Cohort study — For matched studies, give matching criteria and number of exposed and unexposed | **N/A** | No matching performed |
| 7 | Clearly define all outcomes, exposures, predictors, potential confounders, and effect modifiers. Give diagnostic criteria, if applicable | **Yes** | Section 2.3 (Definitions) |
| 8 | For each variable of interest, give sources of data and details of methods of assessment (measurement). Describe comparability of assessment methods if there is more than one group | **Yes** | Sections 2.3, 2.4 |
| 9 | Describe any efforts to address potential sources of bias | **Yes** | Sections 2.1, 2.5; Discussion (Limitations) |
| 10 | Explain how the study size was arrived at | **Yes** | Section 2.1 (all eligible insertions included; census design) |
| 11 | Explain how quantitative variables were handled in the analyses. If applicable, describe which groupings were chosen and why | **Yes** | Section 2.5 (Statistical Analysis) |
| 12(a) | Describe all statistical methods, including those used to control for confounding | **Yes** | Section 2.5 |
| 12(b) | Describe any methods used to examine subgroups and interactions | **Yes** | Section 2.5 (sensitivity analyses, cross-stratification) |
| 12(c) | Explain how missing data were addressed | **Yes** | Section 2.5 (missing data statement) |
| 12(d) | Cohort study — If applicable, explain how loss to follow-up was addressed | **Yes** | Section 2.5; S3 Table (in-situ/LTFU) |
| 12(e) | Describe any sensitivity analyses | **Yes** | Section 2.5; Section 3.9 |
| **Results** | | | |
| 13(a) | Report numbers of individuals at each stage of study — e.g. numbers potentially eligible, examined for eligibility, confirmed eligible, included in the study, completing follow-up, and analysed | **Yes** | Section 3.1; Figure 1 (flowchart) |
| 13(b) | Give reasons for non-participation at each stage | **Yes** | Section 2.4 (exclusion criteria); Figure 1 |
| 13(c) | Consider use of a flow diagram | **Yes** | Figure 1 |
| 14(a) | Give characteristics of study participants (e.g. demographic, clinical, social) and information on exposures and potential confounders | **Yes** | Table 1; Section 3.2 |
| 14(b) | Indicate number of participants with missing data for each variable of interest | **Yes** | Section 2.5 (1 missing outcome, 3 missing INR) |
| 14(c) | Cohort study — Summarise follow-up time (e.g. average and total amount) | **Yes** | Table 1 (total catheter-days); Section 2.3 (dwell time definition) |
| 15 | Report numbers of outcome events or summary measures over time | **Yes** | Sections 3.4–3.5; Tables 1, 3, 4 |
| 16(a) | Give unadjusted estimates and, if applicable, confounder-adjusted estimates and their precision (e.g. 95% CI). Make clear which confounders were adjusted for and why they were included | **Yes** | Sections 3.5, 3.9; Table 4 |
| 16(b) | Report category boundaries when continuous variables were categorised | **Yes** | Section 2.3 (sequence strata: 1st, 2nd, 3rd+); Table 4 |
| 16(c) | If relevant, consider translating estimates of relative risk into absolute risk for a meaningful time period | **Yes** | Section 3.9 (absolute rate increments per sequence step reported) |
| 17 | Report other analyses done — e.g. analyses of subgroups and interactions, and sensitivity analyses | **Yes** | Section 3.9; S1, S2, S3 Tables |
| **Discussion** | | | |
| 18 | Summarise key results with reference to study objectives | **Yes** | Discussion, paragraph 1 (Principal findings) |
| 19 | Discuss limitations of the study, taking into account sources of potential bias or imprecision. Discuss both direction and magnitude of any potential bias | **Yes** | Discussion (Limitations and future directions) |
| 20 | Give a cautious overall interpretation of results considering objectives, limitations, multiplicity of analyses, results from similar studies, and other relevant evidence | **Yes** | Discussion; Conclusion |
| 21 | Discuss the generalisability (external validity) of the study results | **Yes** | Discussion (Limitations, paragraph 1) |
| **Other information** | | | |
| 22 | Give the source of funding and the role of the funders for the present study and, if applicable, for the original study on which the present article is based | **Yes** | Declarations (Funding) |

Note: This checklist is based on the STROBE Statement (von Elm E et al. PLoS Med. 2007;4(10):e296). Items are mapped to the corresponding sections of the submitted manuscript. N/A = not applicable to this study design.
