# Supplementary material for: SEQuential tUnneled Catheters and Infective Events in haemodiaLysis: A Five-Year Retrospective Cohort Study (SEQUEL Study)
Source: PLoS One. 2026 Nov 4;21(11):e0362091. doi: 10.1371/journal.pone.0362091 (PMC13734602; doi:10.1371/journal.pone.0362091)
Supplement: Supplementary file 3 [file pone.0362091.s002.docx]

**S1 Table. CRBSI microbiology and clinical outcomes**

Denominators = number of CRBSI events in the corresponding cohort and site.

| **Microbiology / outcome** | **Index FV (n=18)** | **Index IJV (n=6)** | **All FV (n=103)** | **All IJV (n=51)** |
| --- | --- | --- | --- | --- |
| **Pathogen profile** | | | | |
| Gram positive | 8 (44.4%) | 3 (50.0%) | 49 (47.6%) | 20 (39.2%) |
| Gram negative | 8 (44.4%) | 2 (33.3%) | 41 (39.8%) | 20 (39.2%) |
| Polymicrobial | 0 (0.0%) | 0 (0.0%) | 0 (0.0%) | 0 (0.0%) |
| Culture negative | 2 (11.1%) | 1 (16.7%) | 13 (12.6%) | 10 (19.6%) |
| **CRBSI clinical outcome** | | | | |
| Resolved | 14 (77.8%) | 4 (66.7%) | 62 (60.2%) | 30 (58.8%) |
| Recurrence | 4 (22.2%) | 1 (16.7%) | 33 (32.0%) | 13 (25.5%) |
| Shock / ACS | 0 (0.0%) | 0 (0.0%) | 0 (0.0%) | 0 (0.0%) |
| ICU admission | 0 (0.0%) | 0 (0.0%) | 0 (0.0%) | 0 (0.0%) |
| Death | 0 (0.0%) | 1 (16.7%) | 7 (6.8%) | 8 (15.7%) |
| Catheter salvaged | 5 (27.8%) | 1 (16.7%) | 45 (43.7%) | 19 (37.3%) |
