# Supplementary material for: SEQuential tUnneled Catheters and Infective Events in haemodiaLysis: A Five-Year Retrospective Cohort Study (SEQUEL Study)
Source: PLoS One. 2026 Nov 4;21(11):e0362091. doi: 10.1371/journal.pone.0362091 (PMC13734602; doi:10.1371/journal.pone.0362091)
Supplement: Supplementary file 4 [file pone.0362091.s003.docx]

**S2 Table. CRBSI incidence by year of insertion (all-TCC cohort)**

| **Year** | **Insertions (n)** | **CRBSI events** | **Catheter-days** | **Rate per 1000 cath-days** |
| --- | --- | --- | --- | --- |
| 2016 | 55 | 19 | 8821 | 2.15 |
| 2017 | 80 | 22 | 13094 | 1.68 |
| 2018 | 99 | 27 | 18113 | 1.49 |
| 2019 | 109 | 26 | 23596 | 1.10 |
| 2020 | 155 | 41 | 21183 | 1.94 |
| 2021 | 128 | 19 | 13920 | 1.36 |
