# Supplementary material for: SEQuential tUnneled Catheters and Infective Events in haemodiaLysis: A Five-Year Retrospective Cohort Study (SEQUEL Study)
Source: PLoS One. 2026 Nov 4;21(11):e0362091. doi: 10.1371/journal.pone.0362091 (PMC13734602; doi:10.1371/journal.pone.0362091)
Supplement: Supplementary file 5 [file pone.0362091.s004.docx]

**S3 Table. Reason for catheter removal by site and cohort**

Percentages are of catheters in the corresponding cohort×site stratum. Percentages do not sum to 100% because rare removal reasons are combined into an "Other/not shown" category in this table; omitted categories with their combined counts (FV all-TCC / IJV all-TCC) include: insertion-site infection, self-removal, and kinking. Abbreviations: AV matured = arteriovenous access maturation; Cath. thrombus = catheter thrombosis/mechanical failure; Blockage = catheter poor flow or blockage; In-situ / LTFU = catheter remaining in-situ at end of follow-up or lost to follow-up.

| **Cohort × site** | **n** | **CRBSI** | **AV matured** | **Cath. thrombus** | **Migration** | **Blockage** | **In-situ / LTFU** |
| --- | --- | --- | --- | --- | --- | --- | --- |
| Index – FV | 95 | 16 (16.8%) | 26 (27.4%) | 8 (8.4%) | 7 (7.4%) | 8 (8.4%) | 26 (27.4%) |
| Index – IJV | 40 | 6 (15.0%) | 8 (20.0%) | 1 (2.5%) | 4 (10.0%) | 4 (10.0%) | 7 (17.5%) |
| All-TCC – FV | 389 | 81 (20.8%) | 75 (19.3%) | 30 (7.7%) | 28 (7.2%) | 38 (9.8%) | 113 (29.0%) |
| All-TCC – IJV | 237 | 37 (15.6%) | 39 (16.5%) | 14 (5.9%) | 21 (8.9%) | 49 (20.7%) | 45 (19.0%) |
