# Supplementary material for: SEQuential tUnneled Catheters and Infective Events in haemodiaLysis: A Five-Year Retrospective Cohort Study (SEQUEL Study)
Source: PLoS One. 2026 Nov 4;21(11):e0362091. doi: 10.1371/journal.pone.0362091 (PMC13734602; doi:10.1371/journal.pone.0362091)
Supplement: Peer review history

# Peer review: Decision Letter 0

**DOI:** 10.1371/journal.pone.0362091.r001

**Authors:** Haipeng Liu

28 Aug 2026

Dear Dr. Gan,

Thank you for submitting your manuscript to PLOS One. After careful consideration, we feel that it has merit but does not fully meet PLOS One’s publication criteria as it currently stands. Therefore, we invite you to submit a revised version of the manuscript that addresses the points raised during the review process.

Please submit your revised manuscript by Oct 27 2026 11:59PM. If you will need more time than this to complete your revisions, please reply to this message or contact the journal office at plosone@plos.org. When you're ready to submit your revision, log on to https://www.editorialmanager.com/pone/ and select the 'Submissions Needing Revision' folder to locate your manuscript file.

- A letter that responds to each point raised by the academic editor and reviewer(s). You should upload this letter as a separate file labeled 'Response to Reviewers'.
- A marked-up copy of your manuscript that highlights changes made to the original version. You should upload this as a separate file labeled 'Revised Manuscript with Track Changes'.
- An unmarked version of your revised paper without tracked changes. You should upload this as a separate file labeled 'Manuscript'.

If you would like to make changes to your financial disclosure, please include your updated statement in your cover letter. Guidelines for resubmitting your figure files are available below the reviewer comments at the end of this letter.

If applicable, we recommend that you deposit your laboratory protocols in protocols.io to enhance the reproducibility of your results. Protocols.io assigns your protocol its own identifier (DOI) so that it can be cited independently in the future. For instructions see: https://journals.plos.org/plosone/s/submission-guidelines#loc-laboratory-protocols. Additionally, PLOS One offers an option for publishing peer-reviewed Lab Protocol articles, which describe protocols hosted on protocols.io. Read more information on sharing protocols at https://plos.org/protocols?utm_medium=editorial-email&utm_source=authorletters&utm_campaign=protocols.

As the corresponding author, your ORCID iD is verified in the submission system and will appear in the published article. PLOS supports the use of ORCID, and we encourage all coauthors to register for an ORCID iD and use it as well. Please encourage your coauthors to verify their ORCID iD within the submission system before final acceptance, as unverified ORCID iDs will not appear in the published article. *Only*  the individual author can complete the verification step; PLOS staff *cannot*  verify ORCID iDs on behalf of authors.

We look forward to receiving your revised manuscript.

Kind regards,

Haipeng Liu

Academic Editor

PLOS One

Journal Requirements:

When submitting your revision, we need you to address these additional requirements.

1. Please ensure that your manuscript meets PLOS ONE's style requirements, including those for file naming. The PLOS ONE style templates can be found at

https://journals.plos.org/plosone/s/file?id=wjVg/PLOSOne_formatting_sample_main_body.pdf and https://journals.plos.org/plosone/s/file?id=ba62/PLOSOne_formatting_sample_title_authors_affiliations.pdf

2. Your ethics statement should only appear in the Methods section of your manuscript. If your ethics statement is written in any section besides the Methods, please delete it from any other section.

3. In the online submission form, you indicated that the minimal dataset underlying the findings of this study contains patient-level clinical data collected from medical records at Universiti Malaya Medical Centre (UMMC), Kuala Lumpur, Malaysia. Due to ethical and legal restrictions imposed by the Medical Research Ethics Committee (MREC No. 20211117-10778) and in accordance with Malaysian patient data privacy regulations, the raw dataset cannot be made publicly available, as it contains potentially re-identifiable clinical information. De-identified summary data and the statistical analysis scripts used in this study are available upon reasonable request to the corresponding author (ccgan@ummc.edu.my). Researchers seeking access to the data will be required to obtain appropriate institutional and ethics approval prior to data sharing.

All PLOS journals now require all data underlying the findings described in their manuscript to be freely available to other researchers, either 1. In a public repository, 2. Within the manuscript itself, or 3. Uploaded as supplementary information.

This policy applies to all data except where public deposition would breach compliance with the protocol approved by your research ethics board. If your data cannot be made publicly available for ethical or legal reasons (e.g., public availability would compromise patient privacy), please explain your reasons on resubmission and your exemption request will be escalated for approval.

4. Please amend either the abstract on the online submission form (via Edit Submission) or the abstract in the manuscript so that they are identical.

5. Please ensure that you refer to Figure 1, 2 in your text as, if accepted, production will need this reference to link the reader to the figure.

6. We notice that your supplementary [figures/tables] are included in the manuscript file. Please remove them and upload them with the file type 'Supporting Information'. Please ensure that each Supporting Information file has a legend listed in the manuscript after the references list.

7. Please include a copy of Table 3 which you refer to in your text on page 11.

8. If the reviewer comments include a recommendation to cite specific previously published works, please review and evaluate these publications to determine whether they are relevant and should be cited. There is no requirement to cite these works unless the editor has indicated otherwise.

[Note: HTML markup is below. Please do not edit.]

Reviewers' comments:

Reviewer's Responses to Questions

**Comments to the Author**

1. Is the manuscript technically sound, and do the data support the conclusions?

Reviewer #1: Yes

Reviewer #2: Partly

Reviewer #3: Yes

Reviewer #4: Yes

**********

2. Has the statistical analysis been performed appropriately and rigorously?

Reviewer #1: Yes

Reviewer #2: I Don't Know

Reviewer #3: I Don't Know

Reviewer #4: Yes

**********

3. Have the authors made all data underlying the findings in their manuscript fully available?

The PLOS Data policy

Reviewer #1: Yes

Reviewer #2: Yes

Reviewer #3: Yes

Reviewer #4: Yes

**********

4. Is the manuscript presented in an intelligible fashion and written in standard English?

Reviewer #1: Yes

Reviewer #2: Yes

Reviewer #3: Yes

Reviewer #4: Yes

**********

Reviewer #1: 1. Expand methodological detail on sequence assignment, CRBSI adjudication, and handling of missing data.

2. Present sensitivity analyses more prominently and integrate them into the main results narrative.

3. Provide confidence intervals and more detailed breakdowns of incidence rates.

4. Strengthen the discussion by balancing the sequence versus site findings and elaborating on clinical implications.

5. Revise for clarity, consistency, and conciseness, correcting minor language issues.

6. Expand the limitations section to acknowledge residual confounding and generalisability concerns.

The manuscript is clinically important and has strong potential, but requires minor revisions to improve methodological transparency, statistical clarity, and balanced interpretation. Addressing these points will significantly enhance its impact and credibility.

Reviewer #2: The manuscript addresses a clinically important question whether infection risk among patients receiving hemodialysis through tunnelled catheters is more strongly associated with access site or with repeated catheter placements.

1) The catheter sequence number is not necessarily the patient’s true catheter sequence. The authors have excluded patients with any documented TCC insertion before January 2016, which is appropriate. Nevertheless, the resulting catheter remains the patient’s first registry-recorded TCC rather than a definitively verified first-ever lifetime TCC insertion. This is because undocumented or externally performed prior insertion cannot be excluded. The terminology in the manuscript should be changed from ‘first-ever TCC’ to ‘registry-index TCC’

2) The observed sequence association requires further examination, since the ‘sequence effect’ may not be caused by just the sequence itself. A subsequent catheter insertion is frequently done due to preceding catheter developing complications including CRBSI, malfunction, thrombosis, etc. Previous CRBSI and underlying patient susceptibility may therefore predict a higher sequence number and future CRBSI events. The authors should adjust for previous CRBSI, preceding catheter-removal reasons, and previous sites. Until then, catheter sequence should be interpreted as a risk marker and not an independent causal determinant.

3) There is a confounding issue regarding the femoral vs jugular comparison. Almost all the femoral placements had occurred because jugular access was unavailable or the jugular attempt had already failed. Only one femoral catheter was described as an initially planned femoral catheter approach. Therefore, the study does not actually compare similar patients independently assigned to femoral or jugular access. It instead compares jugular access group with a predominantly rescue-access femoral group. Thus, failed jugular attempts, procedure duration, vascular exhaustion all may affect infection risk.

4) The definition of ‘clinically adjudicated probable CRBSI’ definition needs further explanation. What was the diagnostic criteria for these cases. The criteria for culture-negative cases are insufficiently described, and unclear if alternative infection sources were excluded, or prior antibiotics were used.

5) Inconsistencies were noted in the figures. The manuscript reports index-cohort log-rank p-value of 0.435, but figure 4 mentions p value of 0.340.

6) Figure 8 shows the bars with 194 patients with one catheter and 145 with two or more, which is consistent with the text, but the legend says n= 238 and n = 101.

Reviewer #3: The study explores CRBSI in ESKD patients

The study is statistics-heavy.

Simpler language may help.

This is a femoral vein-centric approach, which is not routinely performed in other centers. It has been deployed for failed IJV insertion even in first timers- seems unusual.

What was the exact IDSA criteria used for probable CRBSI?

Any particular reason why femoral catheters did not have GNB as expected?

Suggestion of femoral catheters as an alternative should be with a caveat: DVT has not been addressed

Reviewer #4: Dear Authors

It is a great job.

I have some comments please ;

1- Because you used so many abbreviations , make a list for them

2- Femoral vein cannulation is associated with increasing risk of DVT including Iliac veins and if there is feature plan for kidney transplantation , it may complicated the procedure.

Thank you

**********

what does this mean?). If published, this will include your full peer review and any attached files.

If you choose “no”, your identity will remain anonymous but your review may still be made public.

**Do you want your identity to be public for this peer review?**  For information about this choice, including consent withdrawal, please see our Privacy Policy

Reviewer #1: **Yes:**  Sura Saad Abdullah

Reviewer #2: No

Reviewer #3: No

Reviewer #4: **Yes:**  Abdullah Saeed Abdullah

**********

[NOTE: If reviewer comments were submitted as an attachment file, they will be attached to this email and accessible via the submission site. Please log into your account, locate the manuscript record, and check for the action link "View Attachments". If this link does not appear, there are no attachment files.]

To ensure your figures meet our technical requirements, please review our figure guidelines: https://journals.plos.org/plosone/s/figures

You may also use PLOS’s free figure tool, NAAS, to help you prepare publication quality figures: https://journals.plos.org/plosone/s/figures#loc-tools-for-figure-preparation.

NAAS will assess whether your figures meet our technical requirements by comparing each figure against our figure specifications.

# Author comment: Author response to Decision Letter 1

**DOI:** 10.1371/journal.pone.0362091.r002

31 Aug 2026

Dear Academic Editor and Reviewers,

We thank you for your careful and constructive comments. We have revised the manuscript substantially and uploaded a detailed point-by-point Response to Reviewers document, a clean revised manuscript, and a revised manuscript with tracked changes.

In response to the editorial requirements, we reformatted the manuscript, placed the ethics statement solely in the Methods section, revised the Data Availability Statement, ensured that the online abstract matches the manuscript, added explicit in-text citations to Figures 1 and 2, removed supplementary materials from the main manuscript and uploaded them separately as Supporting Information, added the previously missing Table 3, and included Supporting Information legends after the references.

In response to the reviewers, we clarified that the index catheter is a registry-index TCC rather than a definitively first-ever lifetime catheter; expanded the operational definition and adjudication process for clinically adjudicated probable CRBSI; clarified missing-data handling; added incidence-density confidence intervals; corrected the Figure 4 log-rank p-value and Figure 8 legend; quantified the predominantly rescue-access nature of femoral TCC placement; added an extended sensitivity Cox model adjusting for previous CRBSI and previous CRBSI-related catheter removal; and strengthened discussion of residual confounding, DVT, femoral/iliac venous complications, and considerations for future kidney transplantation.

We believe these revisions address all editorial and reviewer comments and have improved the clarity, methodological transparency, and balance of the manuscript.

Sincerely,

Chye Chung Gan

Corresponding Author

Attachment Submitted filename: SEQUEL_Response_Letter 1.pdf

# Peer review: Decision Letter 1

**DOI:** 10.1371/journal.pone.0362091.r003

**Authors:** Haipeng Liu

20 Oct 2026

SEQuential tUnneled Catheters and Infective Events in haemodiaLysis: A Five-Year Retrospective Cohort Study (SEQUEL Study)

PONE-D-26-32513R1

Dear Dr. Gan,

We’re pleased to inform you that your manuscript has been judged scientifically suitable for publication and will be formally accepted for publication once it meets all outstanding technical requirements.

Within one week, you’ll receive an e-mail detailing the required amendments. When these have been addressed, you’ll receive a formal acceptance letter and your manuscript will be scheduled for publication.

An invoice will be generated when your article is formally accepted. Please note, if your institution has a publishing partnership with PLOS and your article meets the relevant criteria, all or part of your publication costs will be covered. Please make sure your user information is up-to-date by logging into Editorial Manager at Editorial Manager® and clicking the ‘Update My Information' link at the top of the page. For questions related to billing, please contact billing support.

If your institution or institutions have a press office, please notify them about your upcoming paper to help maximize its impact. If they’ll be preparing press materials, please inform our press team as soon as possible -- no later than 48 hours after receiving the formal acceptance. Your manuscript will remain under strict press embargo until 2 pm Eastern Time on the date of publication. For more information, please contact onepress@plos.org.

Kind regards,

Haipeng Liu

Academic Editor

PLOS One

Additional Editor Comments (optional):

Reviewers' comments:

Reviewer's Responses to Questions

**Comments to the Author**

Reviewer #1: All comments have been addressed

Reviewer #4: All comments have been addressed

**********

2. Is the manuscript technically sound, and do the data support the conclusions?

Reviewer #1: Yes

Reviewer #4: Yes

**********

3. Has the statistical analysis been performed appropriately and rigorously?

Reviewer #1: Yes

Reviewer #4: Yes

**********

4. Have the authors made all data underlying the findings in their manuscript fully available?

The PLOS Data policy

Reviewer #1: Yes

Reviewer #4: Yes

**********

5. Is the manuscript presented in an intelligible fashion and written in standard English?

Reviewer #1: Yes

Reviewer #4: Yes

**********

Reviewer #1: The revised manuscript demonstrates substantial improvement and has addressed the concerns raised in earlier rounds. The methodology is clearly explained, the data presentation is consistent, and the discussion now aligns well with the stated objectives. Ethical standards and publication guidelines appear to be followed, and I see no issues with dual publication. Overall, the article is well‑structured, contributes meaningfully to the field, and is suitable for publication. I recommend acceptance.

Reviewer #4: (No Response)

**********

what does this mean?). If published, this will include your full peer review and any attached files.

If you choose “no”, your identity will remain anonymous but your review may still be made public.

**Do you want your identity to be public for this peer review?**  For information about this choice, including consent withdrawal, please see our Privacy Policy

Reviewer #1: **Yes:**  Sura Saad Abdullah

Reviewer #4: **Yes:**  Abdullah Saeed Abdullah

**********

# Editor report: Acceptance letter

**DOI:** 10.1371/journal.pone.0362091.r004

**Authors:** Haipeng Liu

PONE-D-26-32513R1

PLOS One

Dear Dr. Gan,

I'm pleased to inform you that your manuscript has been deemed suitable for publication in PLOS One. Congratulations! Your manuscript is now being handed over to our production team.

At this stage, our production department will prepare your paper for publication. This includes ensuring the following:

* All references, tables, and figures are properly cited

* All relevant supporting information is included in the manuscript submission,

* There are no issues that prevent the paper from being properly typeset

You will receive further instructions from the production team, including instructions on how to review your proof when it is ready. Please keep in mind that we are working through a large volume of accepted articles, so please give us a few days to review your paper and let you know the next and final steps.

Lastly, if your institution or institutions have a press office, please let them know about your upcoming paper now to help maximize its impact. If they'll be preparing press materials, please inform our press team within the next 48 hours. Your manuscript will remain under strict press embargo until 2 pm Eastern Time on the date of publication. For more information, please contact onepress@plos.org.

You will receive an invoice from PLOS for your publication fee after your manuscript has reached the completed accept phase. If you receive an email requesting payment before acceptance or for any other service, this may be a phishing scheme. Learn how to identify phishing emails and protect your accounts at https://explore.plos.org/phishing.

If we can help with anything else, please email us at customercare@plos.org.

Thank you for submitting your work to PLOS One and supporting open access.

Kind regards,

PLOS One Editorial Office Staff

on behalf of

Dr. Haipeng Liu

Academic Editor

PLOS One
